# Cranial morphology of the orectolobiform shark, *Chiloscyllium punctatum* Müller & Henle, 1838

**DOI:** 10.3897/vz.72.e84732

**Published:** 2022-06-01

**Authors:** Manuel Andreas Staggl, Daniel Abed-Navandi, Jürgen Kriwet

**Affiliations:** 1 Department of Palaeontology, Faculty of Earth Sciences, Geography and Astronomy, Josef-Holaubek-Platz 2, 1090 Vienna, Austria; 2 Haus des Meeres – Aqua Terra Zoo, Fritz-Grünbaum-Platz 1, 1060 Vienna, Austria; 3 Vienna Doctoral School of Ecology and Evolution (VDSEE), University of Vienna, Djerassiplatz 1, 1030 Vienna

**Keywords:** Chiloscyllidae, Chondrichthyes, dissection, Galeomorphii, micro-CT

## Abstract

Elasmobranchs, comprising sharks, skates, and rays, have a long evolutionary history extending back into the Palaeozoic. They are characterized by various unique traits including a predominantly cartilaginous skeleton, superficial prismatic phosphatic layer, and permanent tooth replacement. Moreover, they exhibit a more or less marked sexual dimorphism. Especially the morphology of the chondrocranium and the elements of the whole cranial region of extant and extinct chondrichthyans can provide valuable information about corresponding functions, e.g. the feeding apparatus might reflect the diet of the animals. However, studies on sexual dimorphisms are lacking in orectolobiform sharks, therefore, little is known about possible sexual dimorphic characters in the cranial region in this group. For this reason, we present in this study a comprehensive morphological description of the cranial region of the brownbanded bamboo shark *Chiloscyllium punctatum* Müller & Henle, 1838, with a special focus on its sexual dimorphic characters. Our results reveal clear morphological differences in both sexes of the examined *C. punctatum* specimens, particularly in the chondrocranium and the mandibular arch. The female specimen shows a comparatively more robust and compact morphology of the chondrocranium. This pattern is also evident in the mandibular arch, especially in the palatoquadrate. The present study is the first to describe the morphology of an orectolobiform shark species in detail using both manual dissection and micro-CT data. The resulting data furthermore provide a starting point for pending studies and are intended to be a first step in a series of comparative studies on the morphology of the cranial region of orectolobiform sharks, including the determination of possible sexual dimorphic characteristics.

## 1 Introduction

Living gnathostome vertebrates include two major groups, the Chondrichthyes and Osteichthyes that diverged ca. 455 million years ago in the Ordovician (Grogan and Lund 2004; Grogan et al. 2012). While both groups form well-defined monophyletic groups, the extant members display very distinct skeletons with chondrichthyans having skeletons that consist of a cartilaginous core, which is stiffened by a mineralized collar, while most other living vertebrates have skeletons consisting entirely of bone (Seidel et al. 2020). This is especially evident in the cranium, which superficially is much more complex in osteichthyans comprising endochondral and dermal bones, while the cranium of chondrichthyans only is a mineralized cartilaginous structure called chondrocranium. Nevertheless, there is evidence that an extensive dermal cranial skeleton might represent the plesiomorphic condition for gnathostomes (Giles et al. 2015). This indicates that the presence of a mineralized cartilaginous skeleton in modern chondrichthyans represents a derived condition for these fishes, but also that the adult cranial skeleton of living members thus only has limited potential to decipher the evolution of skeletal structures in, e.g., the gnathostome cranium in general. However, the chondrocranium of both extant and extinct cartilaginous fishes was shown in many studies covering various groups to represent a useful structure to resolve phylogenetic and taxonomic issues (e.g., White 1936; Holmgren 1940, 1941; El-Toubi and Hamdy 1959, 1967; Springer 1964; Gohar and Mazhar 1964; Stehmann 1970; Hulley 1972; Nakaya 1975; Maisey 1982, 1985, 2004; Nishida 1990; McEachran and Miyake 1990; Shirai 1992; Soares and de Carvalho 2020; de Oliveira Lana et al. 2021).

The chondrocranium of both extant and extinct chondrichthyans moreover provides deeper insights into the function such as the feeding apparatus (e.g., Moss 1977; Motta et al. 1997; Wilga et al. 2000, 2016; Dean et al. 2007; Kolmann et al. 2014; Frey et al. 2020). But the taxonomic coverage and thus the availability of detailed cranial morphological information is quite incomplete rendering the usage of these in phylogenetic analyses complicated. For instance, orectolobiform sharks represent the well-established sister group to a clade consisting of lamniforms and carcharhiniforms (e.g., (Compagno 1973; Carvalho 1996; Douady et al. 2003; Naylor et al. 2005; Human et al. 2006; Heinicke et al. 2009; Amaral et al. 2018; Kousteni et al. 2021). The chondrocranium of orectolobiform sharks nonetheless mainly was considered mainly from functional perspectives (e.g., Lowry and Motta 2007; Motta et al. 2002; Wu 1994). Goto (2001) was the only one who used also cranial characters of various orectolobiforms in his study to resolve the intra-relationships within orectolobiforms. Despite this very broad study, no extensive and detailed examination of chondrocranial structures of individual taxa within orectolobiforms has been performed up to now. The goal of the present study therefore is to describe the chondrocranium including the visceral skeleton and pectoral girdle, and related soft-tissues of the brown-banded bamboo shark *Chiloscyllium punctatum* Müller & Henle, 1838 in detail to increase our knowledge about orectolobiform morphology.

Currently, 498 species of sharks are considered valid (Chondrichthyan Tree of Life, 2021), of which 42 species belong to Orectolobiformes that are arranged in seven families. *Chiloscyllium* (seven species) forms together with *Hemiscyllium* (eight species) the family Hemiscyllidae (Kousteni et al. 2021), which is sister to a clade, comprising short-tail nurse sharks nurse sharks (Ginglymostomatidae including *Pseudoginglymostoma brevicaudatum*), whale sharks (Rhincodontidae) and the zebra sharks (Stegostomatidae). Brachaeluridae and Orectolobidae are sister to this clade. The most plesiomorphic group within Orectolobiformes is the Parascyllidae (Naylor et al. 2012a, 2012b). Most orectolobiforms live in or at least associated with coral reefs with the exception of the whale shark (*Rhincodon typus* Smith, 1828), which is epipelagic. Although the many benthic species live in fairly similar environments and occupy similar ecological niches, they exhibit astonishing variances in body size and shape (Goto 2001). While some species of the Parascyllidae only reach a maximum length of 40 cm, *Rhincodon typus* reportedly reaches a maximum size of 1700–2100 cm (Ebert et al. 2021).

The brown-banded bamboo shark *Chiloscyllium punctatum* Müller & Henle, 1838 (Hemiscyllidae) reaches a total length of up to a length of 132 cm, so it is a comparatively large member of the family Hemiscyllidae. This species occurs from eastern India over large parts of Indonesia to northern Australia and southern Japan (Ebert et al. 2021) in water depths from 0 to more than 85 m in tide pools, shallow water lagoons in the intertidal zone, and in reef areas with sandy areas in between. Its diet comprises various invertebrates, such as molluscs and crustaceans, but also smaller, mostly bottom-dwelling fishes (Ebert et al. 2021). The animals seem to be predominantly active at dusk and at night (MS, personal obs.). They often rest in smaller groups huddled together in their hideouts during the day, similar to nurse sharks (Ginglymostomatidae) (MS, pers. obs.).

Chondrichthyan fishes are characterized by abundant sexual dimorphic characters such as the presence of pelvic claspers in males, differing body sizes, dental and cranial morphologies, jaw shape, mouth length and width, or gill positions (e.g. Ellis and Shackley 1995; Kajiura and Tricas 1996; Kajiura et al. 2005; Filiz and Taskavak 2006; Gutteridge and Bennett 2014; Rangel et al. 2016; Berio et al. 2020). Such sexual dimorphic characters have not been studied in detail in orectolobiform sharks. Therefore, possible dimorphic patterns in the chondrocranial morphology also are considered in the present study. The observed differences ultimately will be discussed within a functional or behavioural context.

We dedicate this study to Professor Dr. Wolfgang Maier (Tübingen) on the occasion of his 80^th^ birthday, for his contributions to vertebrate morphology.

## 2 Materials and methods

One female and one male specimen of the brownbanded bamboo shark (*Chiloscyllium punctatum*) were used in this study with a total length of 81.5 cm for the female specimen (EMRG-Chond-A-3) and 79.5 cm for the male specimen (EMRG-Chond-A-4). Both female and the male specimens thus represent adults. Both specimens were born in 2012 in the department of Palaeontology at the University of Vienna. They died in 2019 there naturally and thereafter were stored at the Department of Palaeontology at the University of Vienna in 96% denaturated ethanol. In this study, we focused on the chondrocranium, visceral arches and pectoral girdle and associated soft tissue structures. Consequently, the body posterior to the pectoral girdle was removed and stored separately.

In a first step, both specimens were placed into an alcohol-iodine solution for increasing the contrast of structures. The solution for the male consisted of 50 g iodine per 1000 ml 96% denatured ethanol. To accelerate the contrasting process, the iodine concentration for staining the female was doubled to 100 g iodine per 1000 ml 96%, denatured ethanol. In general, to facilitate intrusion of the solution, the samples were gradually watered before contrasting. For this, the protocol as shown in in, Table 1 was followed.

For distributing the solution regularly through the soft tissues, the specimens were placed in airtight containers on small distance pieces within the staining solution to allow a magnetic stirrer underneath providing constant circulation of the solution. Subsequently, bot specimens were scanned with a VISCOM X8060 NDT micro-CT device to obtain high resolution CT scans. The female was scanned after 147 days after staining started, the male after 172 days. Scan parameters and additional properties for each scan are summarized in Table 2.

The micro-CT scans were processed with the AMIRA® software (ver. 6.1.1) to obtain 3D computer models from the individual image stacks for both specimens. These scans were used to visualise as many structures as possible. However, since micro-CT images might not be able to demonstrate soft-tissues in detail (see below). Therefore, the two specimens additionally were manually dissected and findings were compared to micro-CT scans. For this, the specimens were washed several times with clear water in a first step and then stored in 96% ethanol. Over a period of six weeks, most of the iodine was possible to be removed from the tissues by continuously exchanging the ethanol and exposing the sample to sunlight. After these six weeks, dissection of the two specimens started with carefully subtracting the skin from the underlying tissue to preserve and document superficial structures, such as the ampullae of Lorenzini. Then the ampullae of Lorenzini and the connective tissues were removed in order to clearly distinguish the individual muscle groups. In a next step, individual muscles and ligaments were removed one after the other for identifying the direction and insertion points of individual muscles. Dissection was conducted only on one side of each specimen to retain a reference to the original state.

## 3 Results

### 3.1 Methodological results

As described above, the female specimen was scanned after 147 days and the male specimen after 172 days of iodine staining, respectively. Both micro-CT scans were performed with a resolution of 0.075 mm. Previous studies have shown that in cartilaginous fishes, only mineralized parts of the cartilaginous skeleton, the teeth and the crystalline lenses are visible in unstained specimens (M.S, pers. obs.), while the different soft tissues are difficult to clearly distinguish displaying a more or less uniform grey value in the micro-CT images. The penetration of the iodine staining occurred rather slowly. After the completion of the staining process, the eyes, especially the lenses, and the muscles showed a very light grey tone. For this reason, a lot of iodine was presumably stored in these areas. The micro-CT scans of both specimens were fairly similar in contrast quality with the female specimen being even slightly richer in contrast and more concise. However, the tissues of the female were badly affected by the iodine that almost completely dissociated the skin from the lower tissue layers. This dissociation also is recognizable in the heart muscle and the olfactory epithelium. Manual dissection, conversely, has the advantage to better distinguish soft tissues such as muscles but on the other side is very invasive destroying a lot of potential information that could be maintained in micro-CT scans.

### 3.2 Morphological results

#### 3.2.1 General morphology

**Figure 1:**
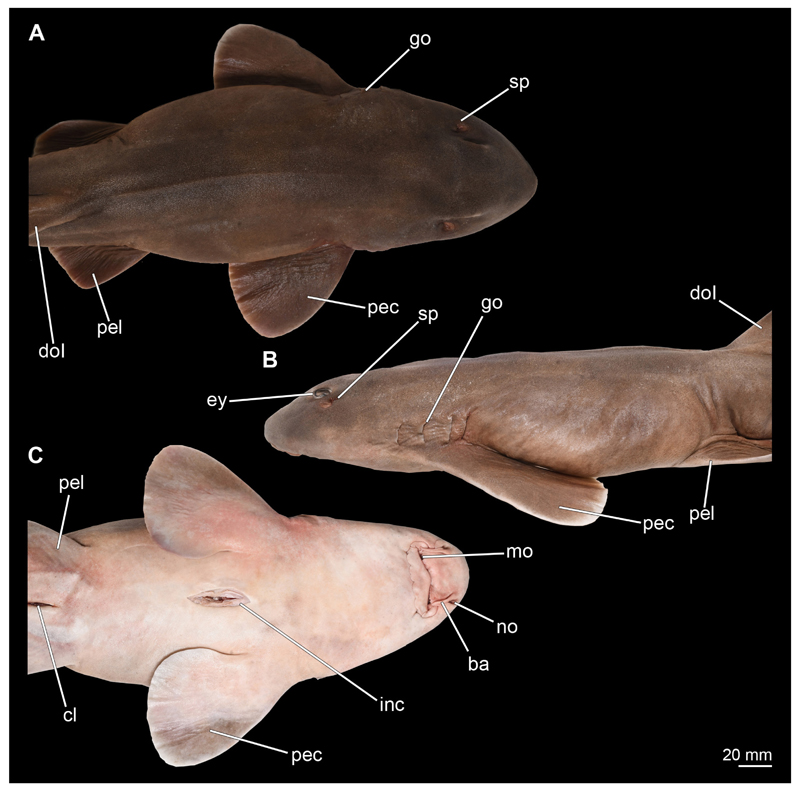
*Chiloscyllium punctatum*, male: Different perspectives of the anterior part of the body up to the pelvic girdle of the male specimen. **A** dorsal, **B** lateral and **C** ventral views. Abbreviations: ba, barbell; cl, cloaca; dol, dorsal fin; ey, eye; go, gill opening; inc, incision; mo, mouth nopening; no, nostril; pec, pectoral fin; pel, pelvic fin; sp, spiracle.

**Figure 2:**
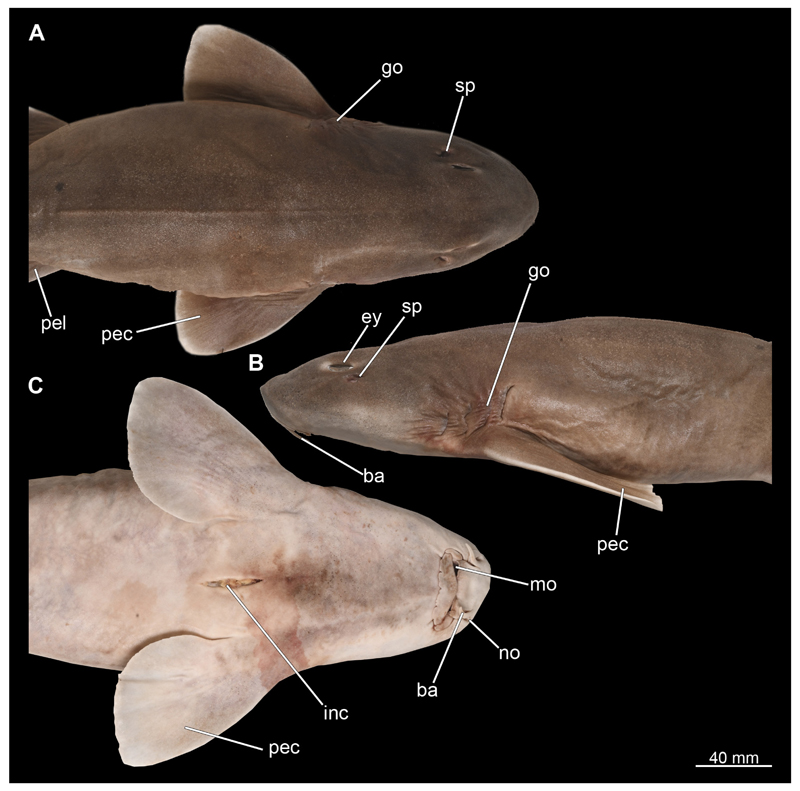
*Chiloscyllium punctatum*, female: Different perspectives of the anterior part of the body up to the pelvic girdle of the female specimen. **A** dorsal, **B** lateral and **C** ventral views. Abbreviations: ba, barbell; cl, cloaca; dol, dorsal fin; ey, eye; go, gill opening; inc, incision; mo, mouth nopening; no, nostril; pec, pectoral fin; pel, pelvic fin; sp, spiracle.

Both specimens of *Chiloscyllium punctatum* **Müller & Henle, 1838** are of a nut-brown colour dorsally, with little to no patterning, while the ventral side is distinctly lighter in colour and creamy white in vivo (Figs 1, 2). The head is spatulate and rather stout, with the male’s head being clearly more acute (Fig. 1A). The female’s head has a much flatter angle anteriorly, which is more arch-like (Fig. 2A). It also is noticeable that, in dorsal view, the head of the male is distinctly broader in the area of the gill openings than that of the female (Fig. 1A). The snout is not very prominent, as is typical for orectolobiform sharks. The width of the snout corresponds approximately to the width of the rest of the head and tapers anteriorly (Figs 1A, 2A). In ventral view, the mouth opening with distinct labial folds, the paired barbels and the nostrils are clearly visible (Figs 1C, 2C). When the mouth is closed, no teeth are visible externally.

In dorsal view, the two spiracles, which are at the same level as the posterior eyelids, are discernible (Figs 1A, 2A). Especially dorsally and ventrally, but also laterally, rows of pores of different sizes are present. They extend from the dorsal side of the rostrum to the lateral side of the head, and from the ventral side to the lateral side ventral to the eyes. The eyes are relatively small in relation to the body and are positioned slightly dorsally on the head (Figs 1A–B and 2A–B). The gill openings are located laterally at the level of the pectoral fins. Four of these gill openings are rather large while the fifth one is noticeably smaller and very closely placed to the fourth one, rendering its identification difficult (Figs 1B, 2B).

#### 3.2.2 Ampullae of Lorenzini

**Figure 3:**
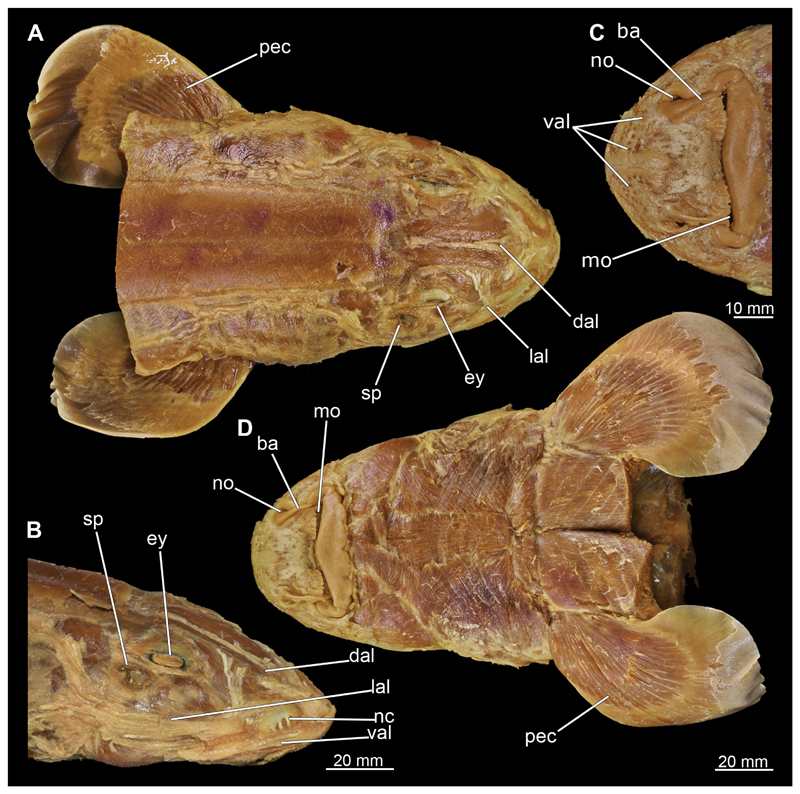
*Chiloscyllium punctatum*, male: Different perspectives of the anterior part of dissected specimen. In this dissection step, only the skin was removed. Large parts of the connective tissue were left on the object. The subcutaneous nerve cords and the ampullae of Lorenzini are well visible. **A** dorsal, **B** lateral, **C** ventral side of the rostrum and **D** ventral views. Abbreviations: ba, barbell; dal, dorsal ampullae of Lorenzini; ey, eye; lal, lateral ampullae of Lorenzini; mo, mouth opening; nc, nasal capsule; no, nostril; pec, pectoral fin; sp, spiracle; val, ventral ampullae of Lorenzini.

**Figure 4:**
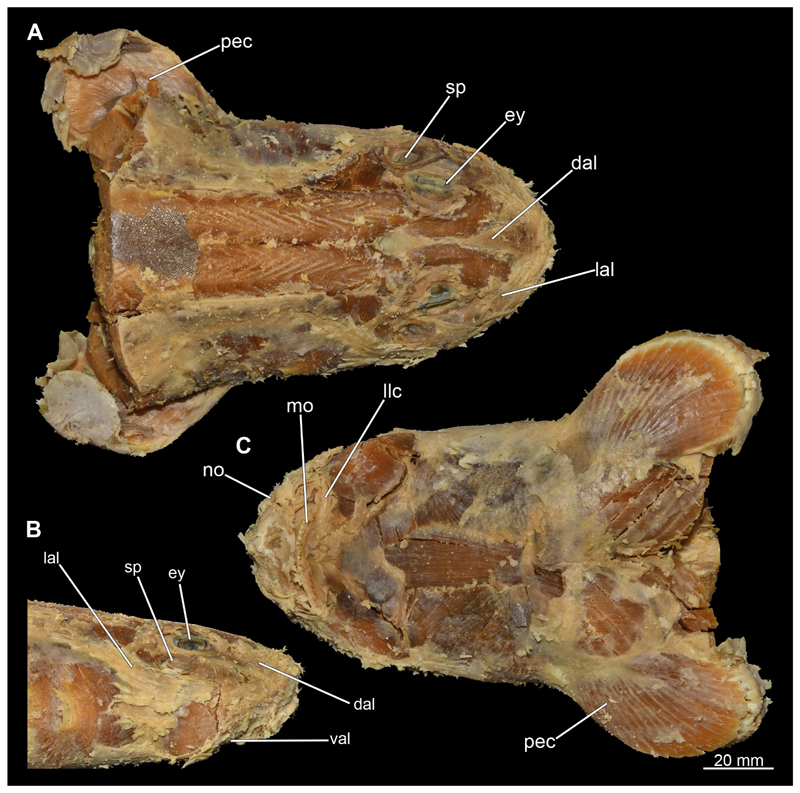
*Chiloscyllium punctatum*, female: Different perspectives of the anterior part of dissected specimen. In this dissection step, only the skin was removed. Large parts of the connective tissue were left on the object. The subcutaneous nerve cords and the ampullae of Lorenzini are well visible. The weak condition of the soft tissues is also evident. **A** dorsal, **B** lateral, **C** ventral views. Abbreviations: ba, barbell; dal, dorsal ampullae of Lorenzini; ey, eye; lal, lateral ampullae of Lorenzini; mo, mouth opening; nc, nasal capsule; no, nostril; pec, pectoral fin; sp, spiracle; val, ventral ampullae of Lorenzini.

After removing the skin during manual dissection, the ampullae of Lorenzini were exposed, particularly in the rostral region (Figs 3, 4). Several individual groups of ampullae can generally be distinguished such as the superficial ophthalmic, the deep ophthalmic, the buccal, the hyoid, and the mandibular ampullae (Goto 2001). The majority of these are located along the dorsal and lateral area of the rostrum. The ducts of the ampullae extend in general from the rostral process to the branchial region as far as the first gill slit with few exceptions that extend posteriorly over the pectoral girdle (Figs 3A–C, 4A–B). Only few ampullae occur ventrally. However, many pores open onto the surface in this area, which correspond to openings of the ampullae, which however not only include those located directly in this area but also those that run laterally along the rostrum. Some ampullae of Lorenzini also are observable dorsally, which run along the chondrocranium between the Musculi levator labii and extend caudally to about the level of the eyes (Figs 3A, 4A).

#### 3.2.3 Chondrocranium

**Figure 5:**
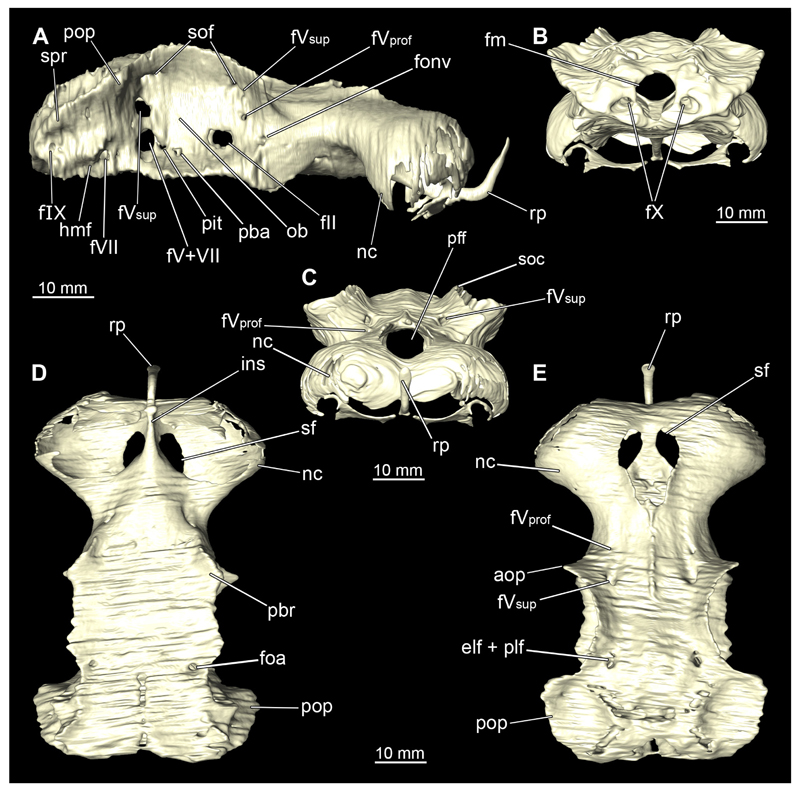
*Chiloscyllium punctatum*, male: Different views of the chondocranium of the specimen. **A** lateral, **B** posterior, **C** anterior, **D** ventral and **E** dorsal views as rendered surface. Abbreviations: aop, Antorbital process; elf, endolymphatic foramen;.fm, foramen magnum; fonv, foramen of orbito-nasal vein; foa, foramen for orbital artery; fII, foramen of cranial nerv II; fV, foramen of cranial nerv V; fVprof, foramen of profundus branch of cranial nerv V; fVsup, foramen of superficial branch of cranial nerv V; fVII, foramen of cranial nerv VII; fIX, foramen of cranial nerv IX; fX, foramen of cranial nerv X; ins, intranasal septum; nc, nasal capsule; ob, orbita; pba, foramen for pseudobranchial artery; pbr, Palatobasal ridge; pff, prefrontal fontanelle; pit, foramen for pituitary vein; plf, perilympatic foramen; pop, postorbital process; rp, rostral process; soc, supraorbital crest; sof, supraorbital foramen; spr, sphenopterotic ridge.

**Figure 6:**
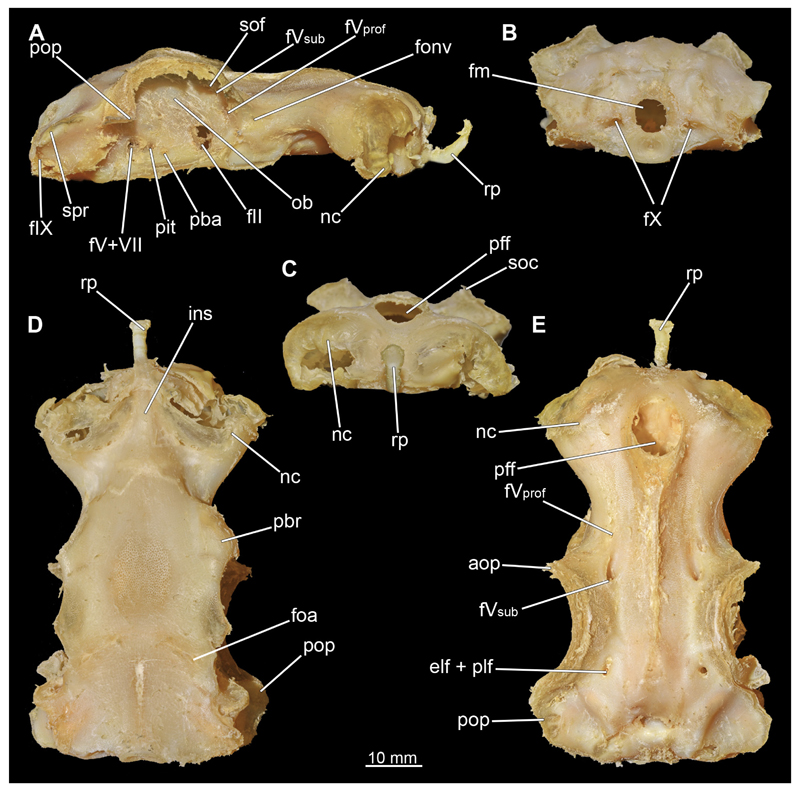
*Chiloscyllium punctatum*, male: Different views of the chondocranium of the dissected specimen. **A** lateral, **B** posterior, **C** anterior, **D** ventral and **E** dorsal views as rendered surface. Abbreviations: aop, Antorbital process; elf, endolymphatic foramen;.fm, foramen magnum; fonv, foramen of orbito-nasal vein; fII, foramen of cranial nerv II; fV, foramen of cranial nerv V; fVprof, foramen of profundus branch of cranial nerv V; fVsup, foramen of superficial branch of cranial nerv V; fVII, foramen of cranial nerv VII; fIX, foramen of cranial nerv IX; fX, foramen of cranial nerv X; ins, intranasal septum; nc, nasal capsule; ob, orbita; pba, foramen for pseudobranchial artery; pbr, Palatobasal ridge; pff, prefrontal fontanelle; pit, foramen for pituitary vein; plf, perilympatic foramen; pop, postorbital process; rp, rostral process; soc, supraorbital crest; sof, supraorbital foramen; spr, sphenopterotic ridge.

**Figure 7:**
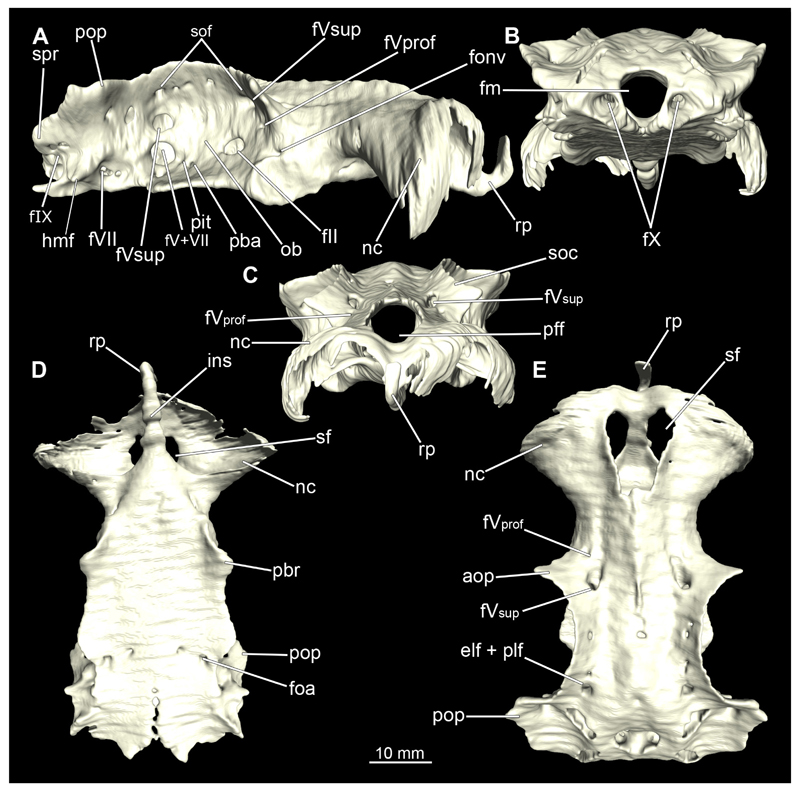
*Chiloscyllium punctatum*, female: Different views of the chondocranium of the specimen. **A** lateral, **B** posterior, **C** anterior, **D** ventral and **E** dorsal views as rendered surface. Abbreviations: aop, Antorbital process; elf, endolymphatic foramen; fm, foramen magnum; fonv, foramen of orbito-nasal vein; foa, foramen for orbital artery; fII, foramen of cranial nerv II; fV, foramen of cranial nerv V; fVprof, foramen of profundus branch of cranial nerv V; fVsup, foramen of superficial branch of cranial nerv V; fVII, foramen of cranial nerv VII; fIX, foramen of cranial nerv IX; fX, foramen of cranial nerv X; ins, intranasal septum; nc, nasal capsule; ob, orbita; pba, foramen for pseudobranchial artery; pbr, Palatobasal ridge; pff, prefrontal fontanelle; pit, foramen for pituitary vein; plf, perilympatic foramen; pop, postorbital process; rp, rostral process; soc, supraorbital crest; sof, supraorbital foramen; spr, sphenopterotic ridge.

**Figure 8:**
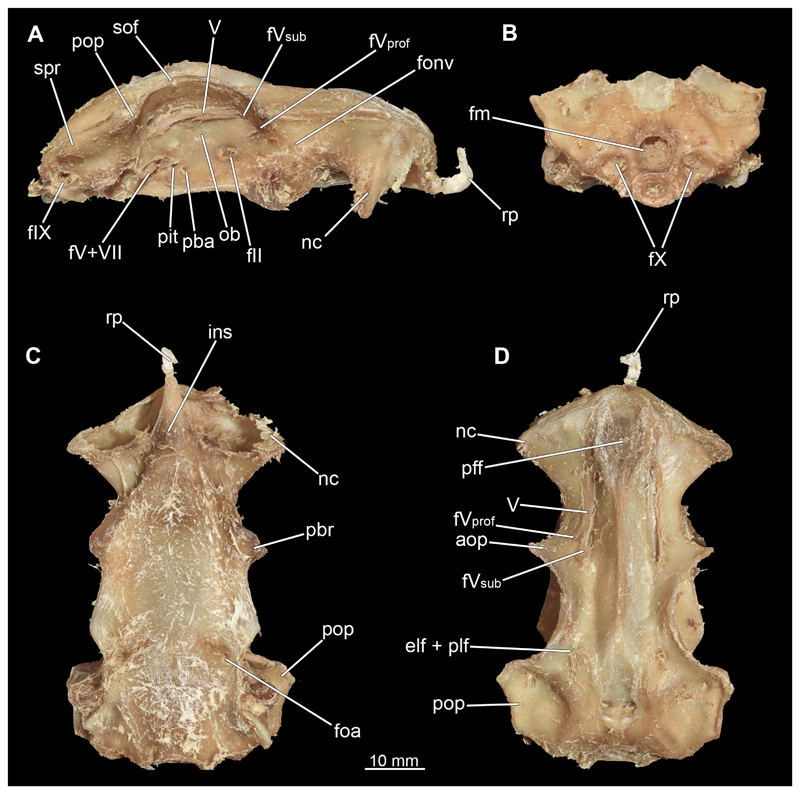
*Chiloscyllium punctatum*, female: Different views of the chondocranium of the dissected specimen. **A** lateral, **B** posterior, **C** anterior, **D** ventral and **E** dorsal views as rendered surface. Abbreviations: aop, Antorbital process; elf, endolymphatic foramen;. fm, foramen magnum; fonv, foramen of orbito-nasal vein; fII, foramen of cranial nerv II; fV, foramen of cranial nerv V; fVprof, foramen of profundus branch of cranial nerv V; fVsup, foramen of superficial branch of cranial nerv V; fVII, foramen of cranial nerv VII; fIX, foramen of cranial nerv IX; fX, foramen of cranial nerv X; ins, intranasal septum; nc, nasal capsule; ob, orbita; pba, foramen for pseudobranchial artery; pbr, Palatobasal ridge; pff, prefrontal fontanelle; pit, foramen for pituitary vein; plf, perilympatic foramen; pop, postorbital process; rp, rostral process; soc, supraorbital crest; sof, supraorbital foramen; spr, sphenopterotic ridge; V, Nervus trigeminalis (CN V).

In general, the chondocranium represents a single large element that encloses and protects both the brain and the sensory organs in this area. This makes it a very distinctive and prominent structure (Figs 5, 6, 7, 8). The nasal capsules are situated relatively distant from the preorbital wall and thus the orbit results in a fairly wide distance between them. The two nasal capsules are separated by a relatively narrow internasal septum, which tapers gradually towards the rostral process (Figs 5D, 6D, 7D, 8C). The subnasal fenestrae are prominent, elliptical (Figs 5D–E, 7D–E) and are directed dorso-ventrally approximately in the middle of the nasal capsules. Ventrally they extend between the preorbital wall and the nasal capsules. The prefrontal fontanel is clearly visible in the scans of both specimens, as well as in both manual dissections (Figs 5C, 6C, E 7C, 8D). The orbital region contains a relatively small eyeball but occupies about one third of the entire skull (Figs 5A, 6A, 7A, 8A).

In lateral view, the first perforation of the skull is that of the orbito-nasal vein. It lies at about the same level (dorso-ventral axis) as the foramen for the optic nerve (CN II), but further anteriorly on the outer edge of the preorbital wall (Figs 5A, 6A, 7A, 8A). Slightly further dorsally are the foramina for both branches of the trigeminal nerve (CN V), the ramus profundus and the ramus superficialis (Fig. 5A, 6A, 7A, 8A). Somewhat more posteriorly, the prominent and very large foramen for the optic nerve (CN II) stands out. This is located at the lower anterior border of the orbita (Figs 5A, 6A, 7A, 8A). Posterior to the optic foramen, the foramen for the pseudobranchial artery and the pituitary vein are located close to each other (Figs 5A, 6A, 7A, 8A). Directly adjacent to this, in the basal area of the orbita, is the prominent foramen for the major branch of the Nervus trigeminalis (CN V) and the Nervus fascialis (CN VII). Directly dorsal to this is another rather large foramen, which is the opening for the ramus superficialis of the Nervus trigeminalis (CN V). Further posteriorly, just anterior to the hyomandibular fossa, the foramen for the fascial nerve is located (CN VII) (Figs 5A, 6A, 7A, 8A).

At the posterior base of the sphenopterotic ridge is the foramen for the glossopharyngial nerve (CN IX) (Figs 5A, 6A, 7A, 8A). This ridge is forming the insertion for the Musculus epaxialis. Following the sphenopterotic ridge anteriorly (and slightly dorsally), the postorbital process, which merges into the supraorbital crest, is situated (Figs 5A, 6A, 7A, 8A). In this crest the superficial ophtalmic foramina opens, a single series of fine perforations running in an arc along the dorsal roof of the orbit (Figs 5A, 6A, 7A, 8A).

In anteriad view, the prominent foramen magnum can be seen and laterally the two foramina for the Nervus vagus (CN X) (Figs 5B, 6B, 7B, 8B). In posteriad view, the foramina for the ramus profundus are well visible and slightly more dorsally those for the ramus superficialis of the Nervus trigeminalis (CN V), both of which perforate at the base of the supraorbital crest (Figs 5B, 6B, 7B).

In dorsal view, the well-developed endolymphatic and perilymphatic fenestrae are exposed. They are located at the level of the posterior end of the orbita (Figs 5E, 6E, 7E, 8D). The antorbital and postorbital processes also are clearly visible (Figs 5E, 6E, 7E, 8D). Ventrally, the palatobasal ridge is at the same level as the antorbital process (Figs 5D, 6D, 7D, 8C). Further posteriorly is the paired foramen for the orbital artery (Figs 5D, 6D, 7D, 8C).

Comparing the micro-CT models of male and female specimens, it is noticeable that the chondrocranium of the female is generally more robustly shaped. The skull of the male, conversely, is proportionally narrower dorso-ventrally. In dorsal view, the male’s chondrocranium is clearly broader, especially when comparing the nasal capsules of both specimens. The distance between the nasal capsule and the orbit is shorter in the female compared to the male. On the other hand, the occipital region, i.e., the area posterior to the orbit, appears to be longer in the female. In the micro-CT model as well as in the dissected skulls, the postorbital processes of the female specimen are more protruding and more angular.

#### 3.2.4 Nasal capsules

**Figure 9:**
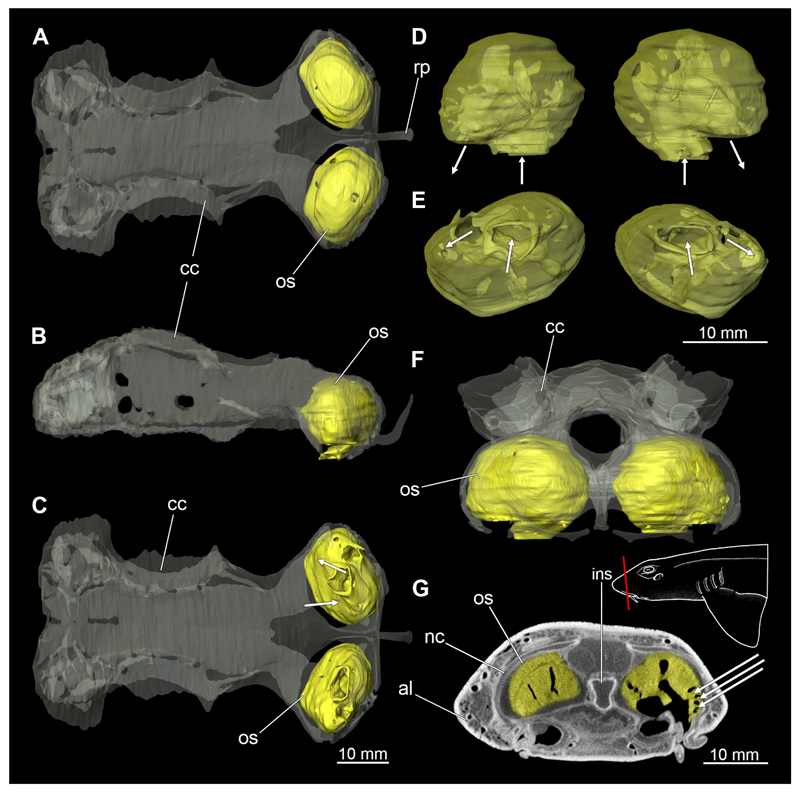
*Chiloscyllium punctatum*, male: Olphactory sacs within the nasal capsules. **A** dorsal, **B** lateral, **C** ventral, arrows indicating direction of waterflow, **D** olphactory sacs anterior, arrows indicating direction of waterflow, **E** olphactory sacs ventral, arrows indicating direction of waterflow, **F** olphactory sacs within chondrocranium anterior and **G** transversal cross section through rostral area of the specimen, yellow markings showing the epithelium, arrows pointing to the folding of the tissue. Abbreviations: al, ampullae of Lorenzini; cc, chondrocranium; ins, intranasal septum; nc, nasal capsules; os, ophactory sacs, rp, rostral process.

**Figure 10:**
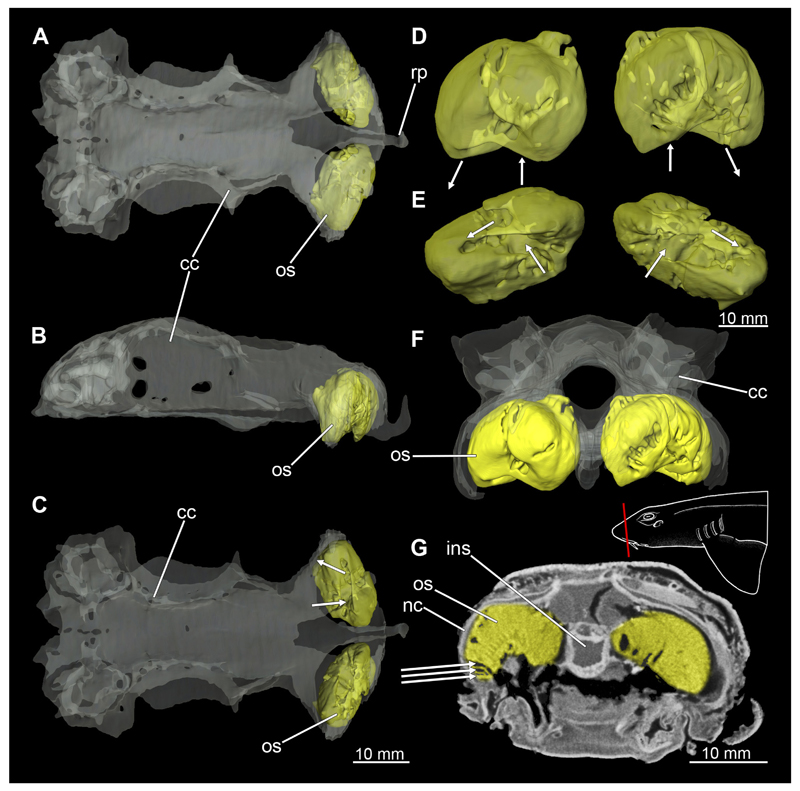
*Chiloscyllium punctatum*, female: Olphactory sacs within the nasal capsules. **A** dorsal, **B** lateral, **C** ventral, arrows indicating direction of waterflow, **D** olphactory sacs anterior, arrows indicating direction of waterflow, **E** olphactory sacs ventral, arrows indicating direction of waterflow, **F** olphactory sacs within chondrocranium anterior and **G** transversal cross section through rostral area of the specimen, yellow markings showing the epithelium, arrows pointing to the folding of the tissue. Abbreviations: al, ampullae of Lorenzini; cc, chondrocranium; ins, intranasal septum; nc, nasal capsules; os, ophactory sacs, rp, rostral process.

The nasal capsules are located anterior to the orbits but are slightly smaller than the orbits. They contain the actual sensory epithelium, which is responsible for olphactoric sensory perception (Figs. 9, 10). This epithelium, called the olphactoric sacs, is roughly shaped like a sphere, with the inner cavity being connected to the nostrils (Goto 2001). Inside, the epithelium is folded up several times, with these folds in turn having creases to increase the surface area. These individual folds can be seen in the micro-CT images in Figure 9 and 10 (Figs. 9G, 10G, arrows point to the folds). These folds are called lamellae, on the surface of which the sensory receptors are located. The olphactoric sacs are directly connected caudally to the olfactory bulbs, which are responsible for transmitting stimuli from the sensory receptors to the olfactory lobes in the brain. The nasal capsules have no connection to the oral cavity. In order to optimise the flow of water through the sensory epithelium, there is an inflow and outflow opening (Figs. 9D–E, 10D–E, arrows mark the direction of flow). The nasal capsules of the female generally appear to be slightly smaller, but this could also be an artefact due to fixation.

#### 3.2.5 Inner ear

**Figure 11:**
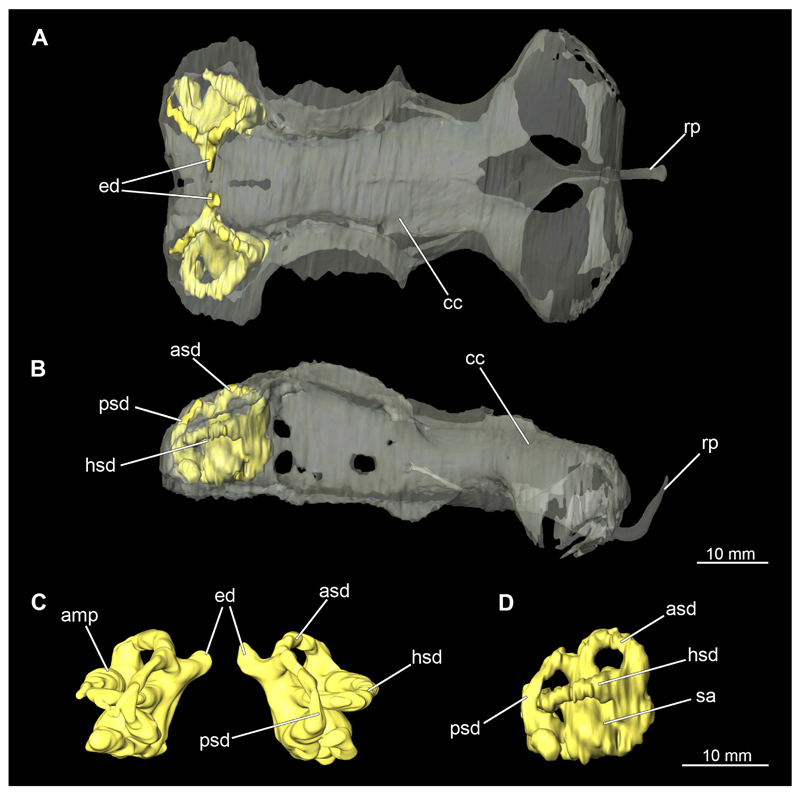
*Chiloscyllium punctatum*, male: Different views of the inner ear of the specimen. **A** dorsal view within the chondro-cranium, **B** lateral view within the chondrocranium, **C** posterior view and **D** lateral view as rendered surface. Abbreviations: amp, Ampulla; asd, anterior semicircular canal, cc, chondrocranium; ed, endolymphatic duct; hsd, horizontal semicircular canal, psd, posterior semicircular canal, rp, rostral process; sa, sacculus.

The inner ear is very prominent in both sexes. However, creating accurate models based on the micro-CT scans is not an easy task, as the transitions to the cerebral cavity are almost fluent, as is the transition to the outer area via the endolymphatic duct. Nevertheless, most of the essential components are clearly visible in both models (Figs 10 and 11). In posterior view, the endolymphatic duct stands out proximally (Figs 10C, 11C). At its base two ducts are situated representing the anterior and the posterior semicircular ducts, respectively. Between these two ducts, the horizontal semicircular duct runs in a semi-curve from anterio-posteriorly. At the base of each duct is a thickening, which has been representatively labelled on the horizontal semicircular duct in Figs 10C, 11C. In lateral view, the sacculus of the inner ear can be seen at the distal side at the base (Figs 10D, 11D). In the micro-CT scan, no otoliths were visible in either specimen, which might have been dissolved, as otoliths should have been present (Schnetz et al. 2019).

#### 3.2.6 Visceral arches

The visceral- or splanchnocranium of *Chiloscyllium punctatum* consists of seven visceral arches, which represent the mandibular arch with associated labial cartilages, the hyoid arch and five branchial arches. Each arch consists of different cartilaginous elements.

##### 3.2.6.1 Mandibular arch

**Figure 13:**
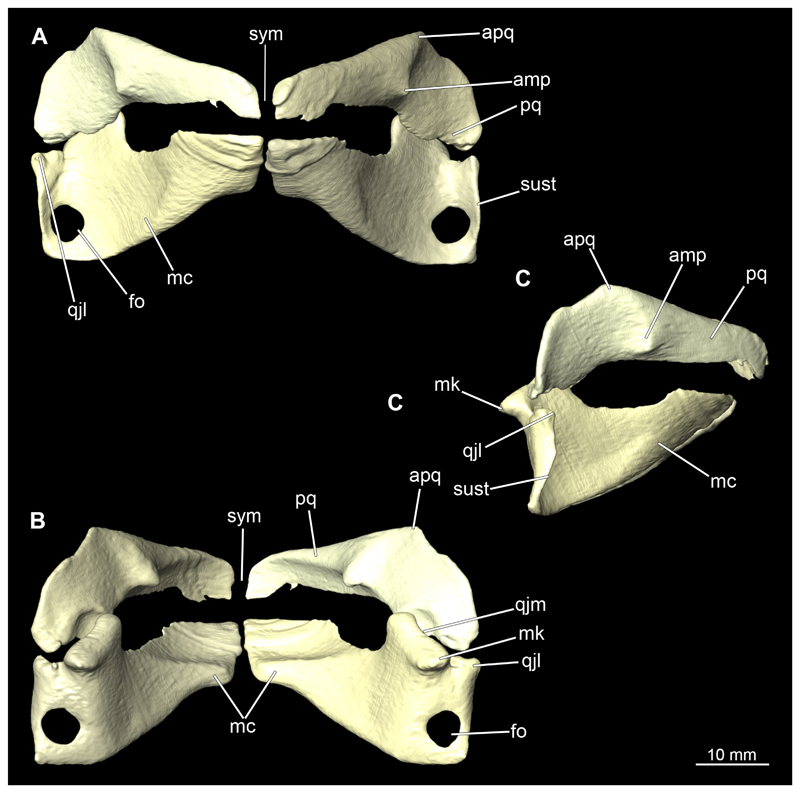
*Chiloscyllium punctatum*, male: Different views of the mandibular arch of the specimen. **A** anterior, **B** posterior and **C** lateral views as rendered surface. Abbreviations: amp, adductor mandibular process; apq, ascending process of the palatoquadratum; fo, foramen; mc, Meckel’s cartilage; mk, mandibular knob; pq, palatoquadratum; qjl, lateral quadratomandibular joint; qjm, medial quadratomandibular joint; sust, sustentaculum; sym, symphysis.

**Figure 14:**
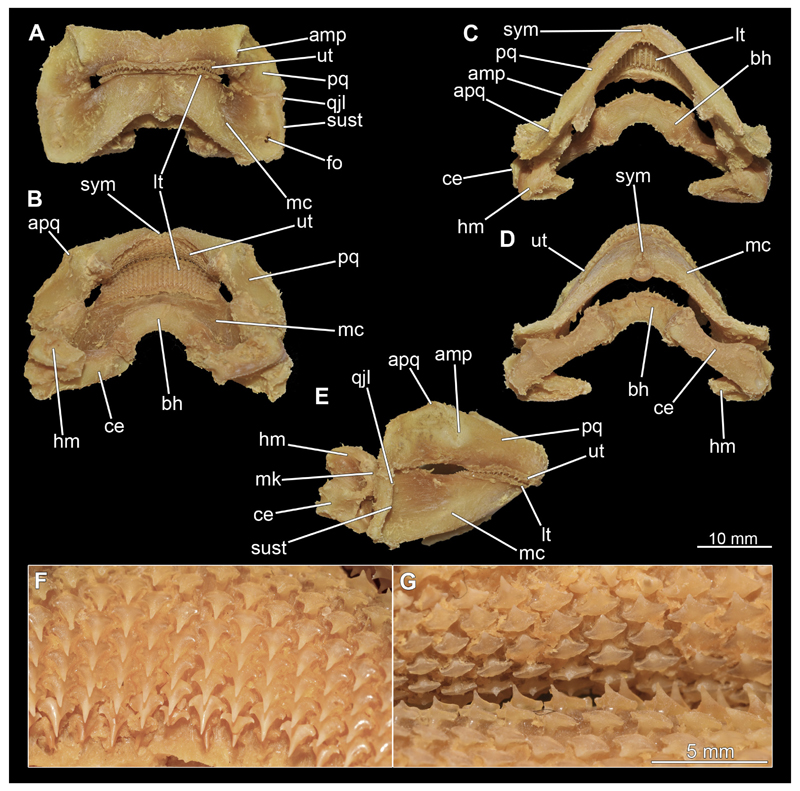
*Chiloscyllium punctatum*, male: Different views of the mandibular and the hyoid arch of the dissected specimen. **A** anterior, **B** posterior, **C** dorsal, **D** ventral and **E** lateral views of the first and second arch of the splanchiocranium, **F** lower teeth, and **G** upper and lower teeth. Abbreviations: amp, adductor mandibular process; apq, ascending process of the palatoquadratum; bh, basihyale; ce, ceratohyal; fo, foramen; hm, hyomandibula; lt, lower teeth; mc, Meckel’s cartilage; mk, mandibular knob; pq, palatoquadratum; qjl, lateral quadratomandibular joint; qjm, medial quadratomandibular joint; sust, sustentaculum; sym, symphysis; ut, upper teeth.

**Figure 15:**
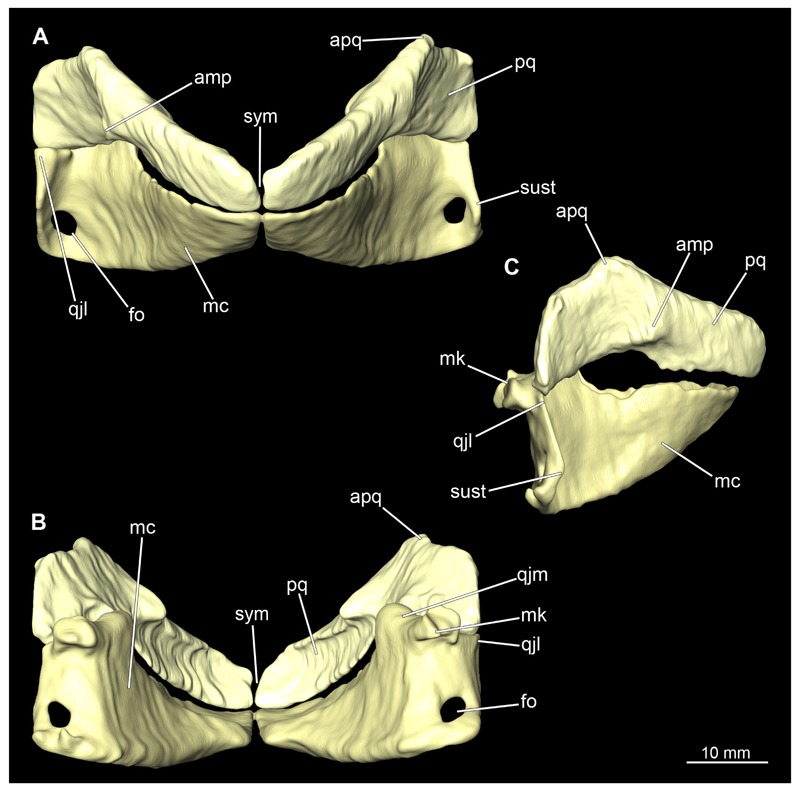
*Chiloscyllium punctatum*, female: Different views of the mandibular arch of the specimen. **A** anterior, **B** posterior and **C** lateral views as rendered surface. Abbreviations: amp, adductor mandibular process; apq, ascending process of the palatoquadratum; fo, foramen; mc, Meckel’s cartilage; mk, mandibular knob; pq, palatoquadratum; qjl, lateral quadratomandibular joint; qjm, medial quadratomandibular joint: sust, sustentaculum; sym, symphysis;

**Figure 16:**
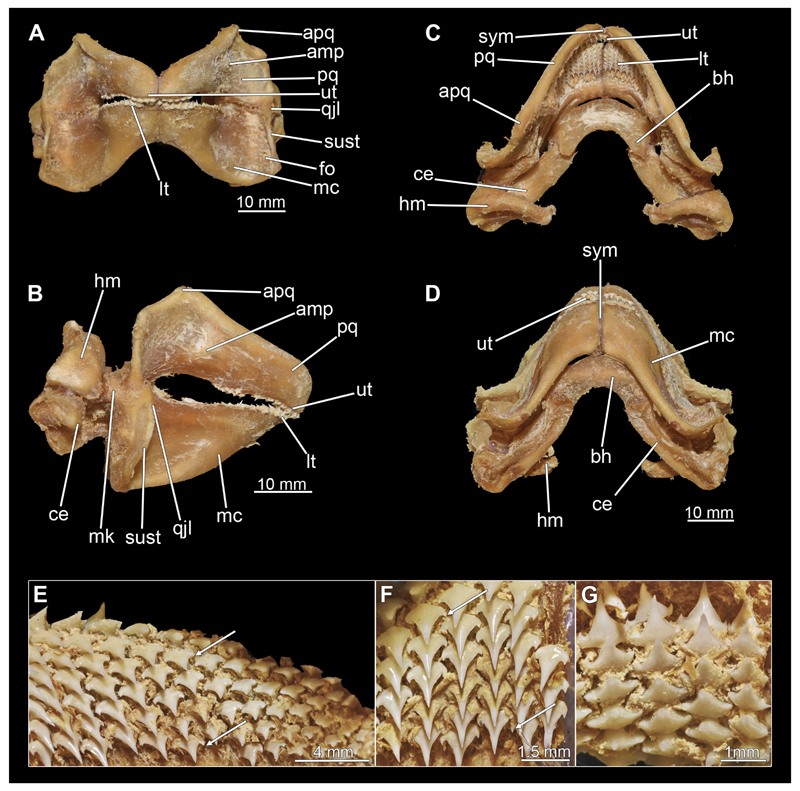
*Chiloscyllium punctatum*, female: Different views of the mandibular and the hyoid arch of the dissected specimen. **A** anterior, **B** lateral, **C** dorsal and **D** ventral views of the first and second arch of the splanchiocranium, **E** lower teeth lateral (arrows pointing to the cusplets), **F** lower teeth anteriat (arrows pointing to the cusplets) and **G** upper teeth anteriat. Abbreviations: amp, adductor mandibular process; apq, ascending process of the palatoquadratum; bh, basihyale; ce, ceratohyal; fo, foramen; hm, hyomandibula; lt, lower teeth; mc, Meckel’s cartilage; mk, mandibular knob; pq, palatoquadratum; qjl, lateral quadratomandibular joint; qjm, medial quadratomandibular joint; sust, sustentaculum; sym, symphysis; ut, upper teeth.

The mandibular arch is composed of a ventral element, the Meckel’s cartilage and a dorsal element, the palatoquadratum (Figs 13, 14, 15, 16). The palatoquadratum represents a rather prominent cartilaginous structure (Figs 13A,C, 14A–B, E, 15A,C, 16A–B). Both antimeres of this cartilage are connected medially by soft tissue along the symphysis (Figs 13A–B, 14B–C, 15A–B, 16C). In lateral view, the palatoquadratum roughly describes a semicircular arch and tapers anteriorly. Centrally, there is a raised process pointing laterally the so called adductor mandibular process (Figs 13C, 14E, 15C, 16B). This serves as the attachment point of the Musculus adductor mandibulae. Dorsally, there is another process, which is directed dorsally. This is the so-called ascending process of the palatoquadratum (Figs 13, 14B–C, E, 15, 16A–C). On the median side of this process there is another protrusion pointing proximally, which serves as the attachment point for the inner quadrato-mandibular ligament.

The dentition of the upper jaw extends medially from the symphysis laterally at about the level of the adductor mandibular process. At this point, the ventral edge of the palatoquadratum describes a dorsal curve and, together with the corresponding ventrally directed Meckel’s cartilage, creates an ellipsoidal gap between the two jaws (Figs 13C, 14E, 15C, 16B). The teeth in the upper jaw are arranged in rows and files. The individual files run from labial to lingual (Figs 14B,G, 16C,G). A file of symphyseal teeth is present medially but does not differ morphologically from the adjacent rows of teeth. Each tooth consists of a pointed, triangular main cusp, which is laterally accompanied at the base by two cusplets (Figs 14G, 16G). The two functional rows of teeth are located along the outer margin of the upper jaw. The replacement teeth develop close to the cartilage with the tip pointing dorsally and turn ventrally over time when the previous tooth reaches the outer edge of the jaw cartilage and is pushed outwards (Figs 14G, 16G). The degree of abrasion, especially of the cusplets, is striking in older teeth compared to younger ones (Figs. 14G, 16G). From anterior to posterior the teeth become noticeably smaller. The rear-most four files of teeth are distinctly different in that they have significantly lower crowns than the anterior ones. The cusplets also are distinctively smaller or even absent. The tooth crowns in the last two files are very reduced forming small hump-like structures.

The Meckel’s cartilage is a distinctly more planar and slender cartilage element than the palatoquadrate (Figs 13, 14A,E, 15, 16A–B). It is also composed of two single elements, which are connected medially along the symphysis by soft tissue (Figs 13A–B, 14D, 15A–B, 16D). In lateral view, the Meckel’s cartilage has an approximately triangular outline. On the dorsal edge, the recess for the ellipsoid shaped gap formed with the upper jaw, as already mentioned for the palatoquadrate, is noticeable. The caudal end of Meckel’s cartilage describes an arc and forms a fold, causing the distal end to face forward. This fold is called the sustentaculum and represents a mechanical reinforcement of the jaw (Figs 13C, 14E, 15C, 16B). At the ventral base of the sustentaculum is a large, circular foramen (Figs 13A–B, 14A, 15A–B, 16A). This is clearly visible in the micro-CT images, but barely noticeable during dissection. The opening appears to be covered by fibrous connective tissue, which is why it is barely visible, if at all. At the posterior edge of the sustentaculum lies the lateral quadrato-mandibular joint, at which the palatoquadrate articulates with the Meckel’s cartilage and thus forms the primary jaw articulation. Functionally, this articulation represents a hinge joint. Posterior to this, at the caudo-lateral end of the Meckel’s cartilage, a process, the mandibular knob, is directed posteriorly. It is a rather flat socket distally, on which the hyomandibula articulates (Figs 13B–C, 14E, 15B–C, 16B) forming the hyomandibulo-mandibular joint.

The teeth of the lower jaw are morphologically very similar to those of the upper jaw, just a little bit sturdier (Figs 14B,F, 16C,E–F). However, there are noticeably more tooth rows in the lower jaw, of which the two labial-most rows represent functional teeth. The apices of the teeth of the other series are directed lingually and represent replacement teeth (Figs 14F, 16E–F). As in the upper jaw, the morphology of the teeth in the distal-most files changes noticeably. Mesio-distally, the teeth decrease noticeably in size along the rows. Especially the teeth in the posterior-most six files are distinctly different. Their apices are considerably lower than those of the anterior teeth. Thus, the teeth are clearly broader than high. The cusplets are also much less distinct and less pronounced. The last few teeth are merely small cusps (Fig. 16E).

**Figure 17:**
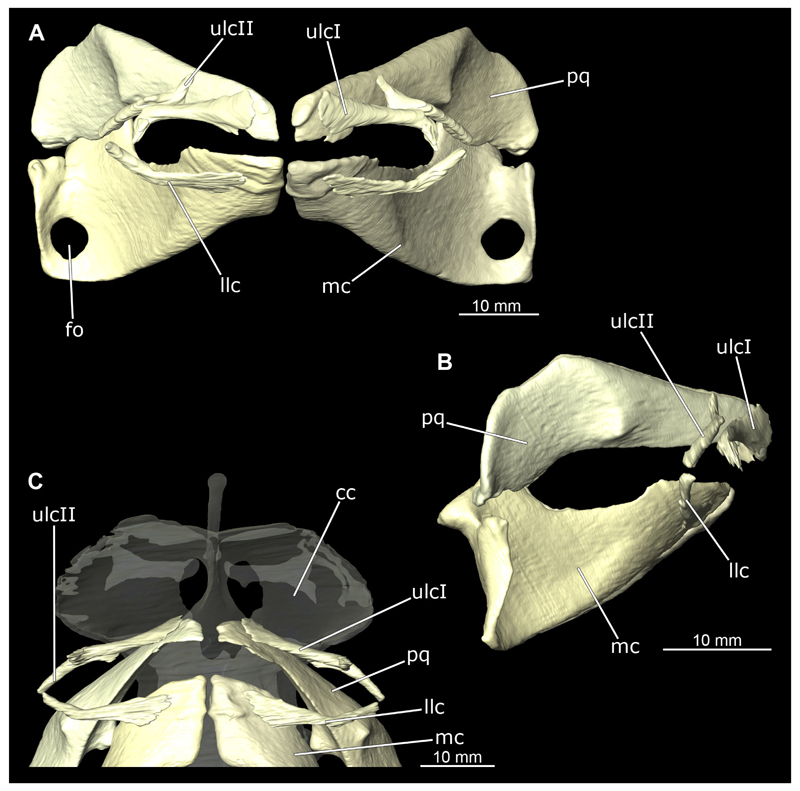
*Chiloscyllium punctatum*, male: Different views of the labial cartilage attached to the mandibular arch of the specimen. **A** anterior, **B** lateral and **C** ventral views as rendered surface. Abbreviations: cc, chondrocranium; fo, foramen; llc, lower labial cartilage; mc, Meckel’s cartilage; pq, palatoquadratum; ulcI, upper labial cartilage I; ulcII, upper labial cartilage II.

**Figure 18:**
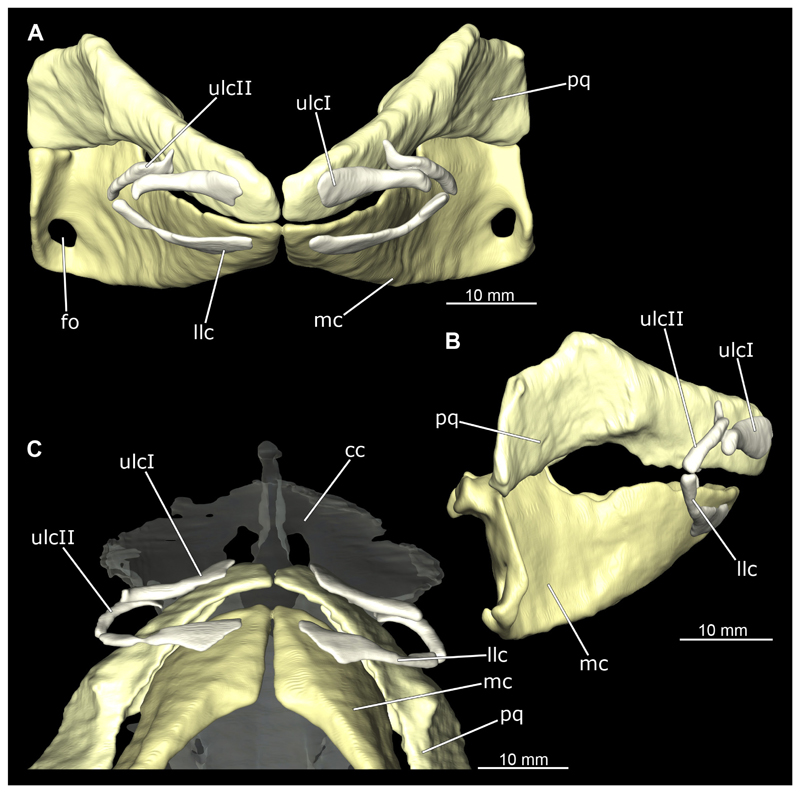
*Chiloscyllium punctatum*, female: Different views of the labial cartilage attached to the mandibular arch of the specimen. **A** anterior, **B** lateral and **C** ventral views as rendered surface. Abbreviations: cc, chondrocranium; fo, foramen; llc, lower labial cartilage; mc, Meckel’s cartilage; pq, palatoquadratum; ulcl, upper labial cartilage I; ulcll, upper labial cartilage II.

In the upper jaw there is a pair of labial cartilages on each side, which supports the upper lip (Figs 17, 18). The first labial cartilage starts very anteriorly, initially runs parallel to the palatoquadratum and then describes an arc ventrally. The second labial cartilage starts slightly more posteriorly, but is morphologically similar to the first one. The lower jaw displays only one labial cartilage (Figs 17, 18). It starts at the same level as the second labial cartilage of the upper jaw. Analogous to the upper jaw, the labial cartilage of the lower jaw runs in an arc dorsally. There, both labial cartilages are almost contacting each other and are connected by soft tissue (Figs. 17, 18).

When comparing both sexes, it is noticeable that the jaw of the female seems to be much more robust. In general, the palatoquadrate and Meckel’s cartilage are more compressed along the anterior-posterior axis compared to the male. Thus, both cartilage elements are clearly higher and therefore more planar than those of the male. The palatoquadrate of the male is relatively narrow, has a rather straight labial edge and curves at the end ventrally, terminating in a hook-like shape. The palatoquadrate of the female remains almost straight and terminates in a rounded edge. The adductor mandibular process of the female palatoquadrate starts slightly more anteriorly but does not differ in relative size from that of the male. The ascending process of the palatoquadrate of the female is more pronounced and dorsally higher. Due to the different shape of the two jaw elements between the sexes, there is also a different shape of the lateral gap formed by the palatoquadrate and Meckel’s cartilage, which is relatively narrow and elongated in the male specimen, whereas it is much broader in the female specimen (Figs 13C, 14E, 15C, 16B).

##### 3.2.6.2 Hyoid arch

**Figure 19:**
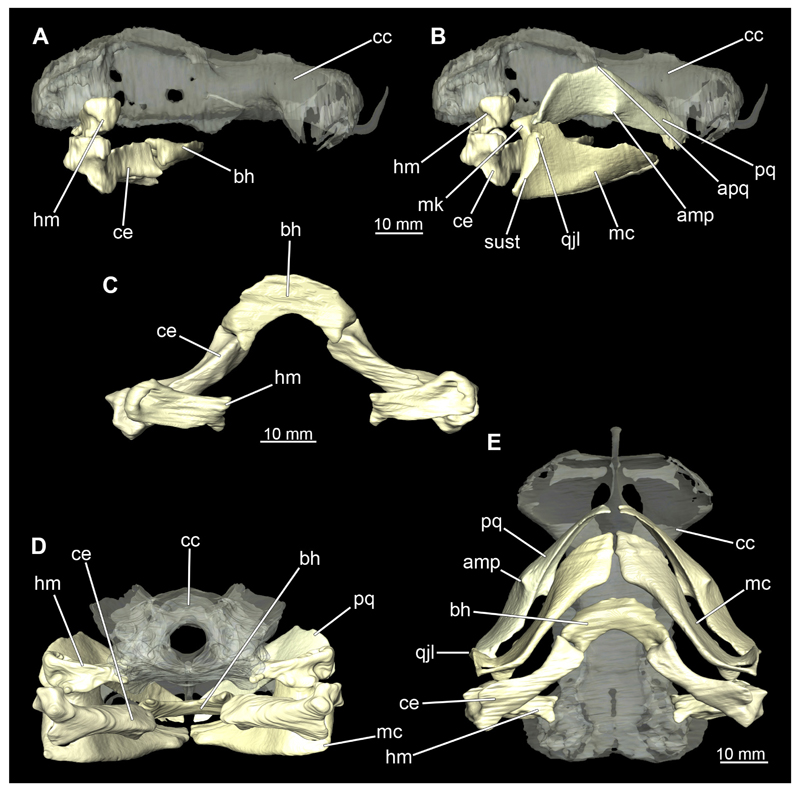
*Chiloscyllium punctatum*, male: Different views of the hyoid and mandibular arch of the specimen. **A** lateral (only hyoid arch), **B** lateral (with mandibular arch), **C** hyoid arch dorsal, **D** posterior and **E** ventral views as rendered surface. Abbreviations: amp, adductor mandibular process; apq, ascending process of the palatoquadratum; bh, basihyale; ce, ceratohyal; cc, chondrocranium; hm, hyomandibula; mc, Meckel’s cartilage; mk, mandibular knob; pq, palatoquadratum; qjl, lateral quadratomandibular joint; sust, sustentaculum; sym, symphysis.

**Figure 20:**
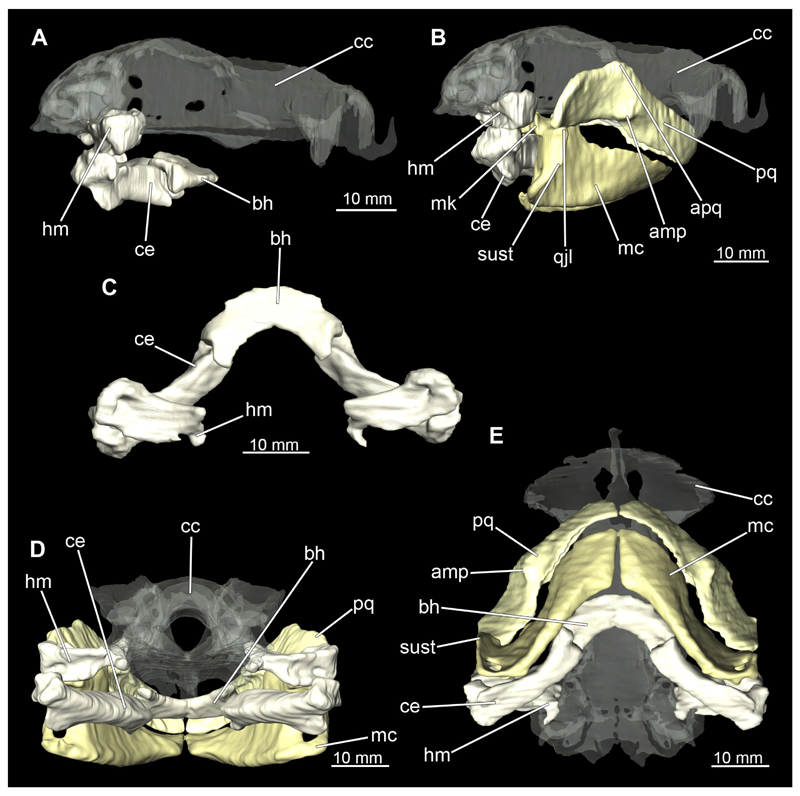
*Chiloscyllium punctatum*, female: Different views of the hyoid and mandibular arch of the specimen. **A** lateral (only hyoid arch), **B** lateral (with mandibular arch), **C** hyoid arch dorsal, **D** posterior and **E** ventral views as rendered surface. Abbreviations: amp, adductor mandibular process; apq, ascending process of the palatoquadratum; bh, basihyale; ce, ceratohyal; cc, chondrocranium; hm, hyomandibula; mc, Meckel’s cartilage; mk, mandibular knob; pq, palatoquadratum; qjl, lateral quadratomandibular joint; sust, sustentaculum; sym, symphysis.

The hyoid arch essentially consists of five elements: One median unpaired basihyal, paired ceratohyales and attached to it, paired hyomandibulae (Figs 14, 16, 19, 20). The ceratohyals and the hyomandibulae are located medial to the jaw elements. The posterior part of the hyomandibula contacts the ceratohyal, which is about one third longer than the hyomandibula. The anterior tips are articulating with the posterior edge of the basihyal (Figs 14B–D, 19C–D, 16C,E, 20C,E).

The basihyal is an unpaired element located at the base of the oral cavity (Figs 14B–D, 19C–D, 16C–E, 20C–E). It is roughly crescent-shaped and curves posteriorly. There are two postero-lateral socket-like facets for articulation with the two ceratohyal. The areas dorsal of these articulation facets are elongated and form rather pointed processes that are pointing backwards. The anterior part of the basihyal is semi-circular as previously described and rests on the coraco-hyoideus muscle (Figs 36B and 38B).

The hyoid arch of the female generally tends to be somewhat more robustly. Other than that, there are no significant differences in the structure of the hyoid arch between the sexes.

##### 3.2.6.3 Branchial arches

**Figure 21:**
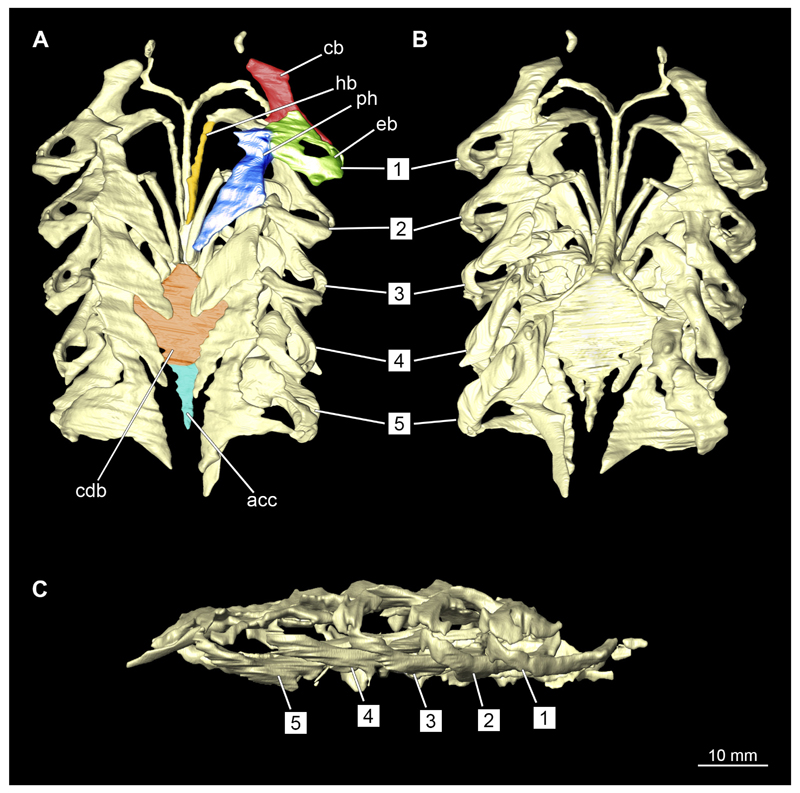
*Chiloscyllium punctatum,* male: Different views of the Visceral arches of the specimen: Abbreviations: **A** dorsal, **B** ventral and **C** lateral right side as rendered surface. Abbreviations: 1–5, visceral arches 1,2,3,4,5; acc, accessory cartilage of basibranchial; bb, basibranchiale; cb, ceratobranchiale; cdb, cardiobranchiale; eb, epibranchiale; hb, hypobranchiale; pb, pharyngo-branchiale.

**Figure 22:**
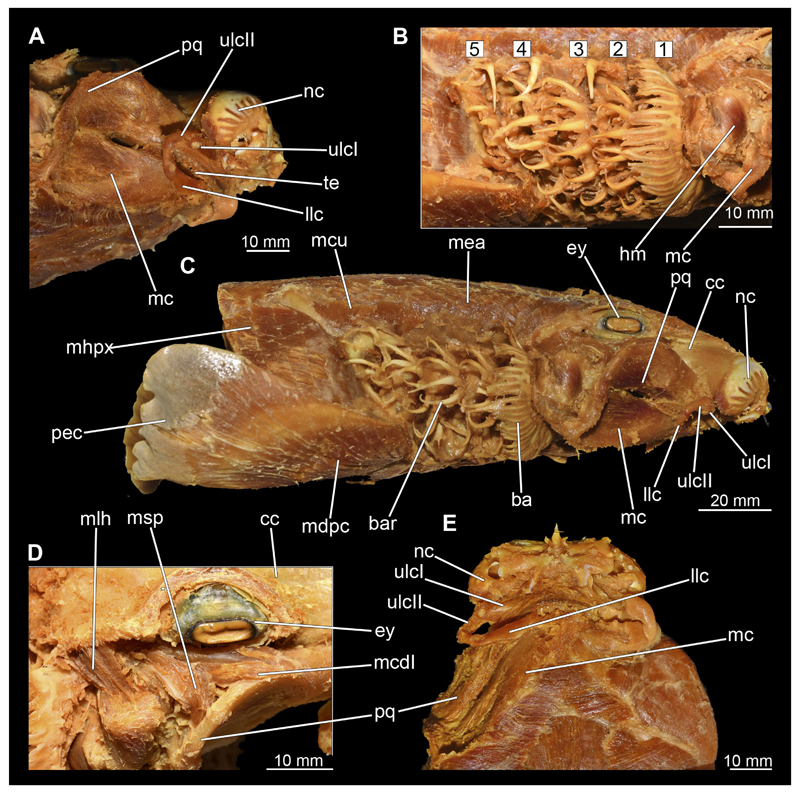
*Chiloscyllium punctatum,* male: Different views of the anterior body part of the specimen with removed epibranchial and mandibular muscles revealing the gill rays, labial cartilages and the single elements of the Musculus constrictor dorsalis. **A** ventro-lateral, **B** detail of the branchial region lateral, **C** lateral, **D** detail M. constrictor dorsalis, dorso-lateral and **E** ventral views of the dissected specimen. Abbreviations: 1–5, visceral arches 1,2,3,4,5; ba, branchial arch; bar, branchial rays; cc, chondrocranium; ey, eye; llc, lower labial cartilage; mc, Meckel’s cartilage; mcdI, musculus constrictor dorsalis I; mcu, musculus cucullaris; mdpc, musculus depressor pectoralis; mea, musculus epiaxialis mhpx, musculus hypaxialis; mlh, musculus levator hyomandibulae; msp, musculus spiracularis; nc, nasal capsule; pec, pectoral fin; pq, palatoquadratum; te, teeth; ulcI, upper labial cartilage I; ulcII, upper labial cartilage II.

**Figure 23:**
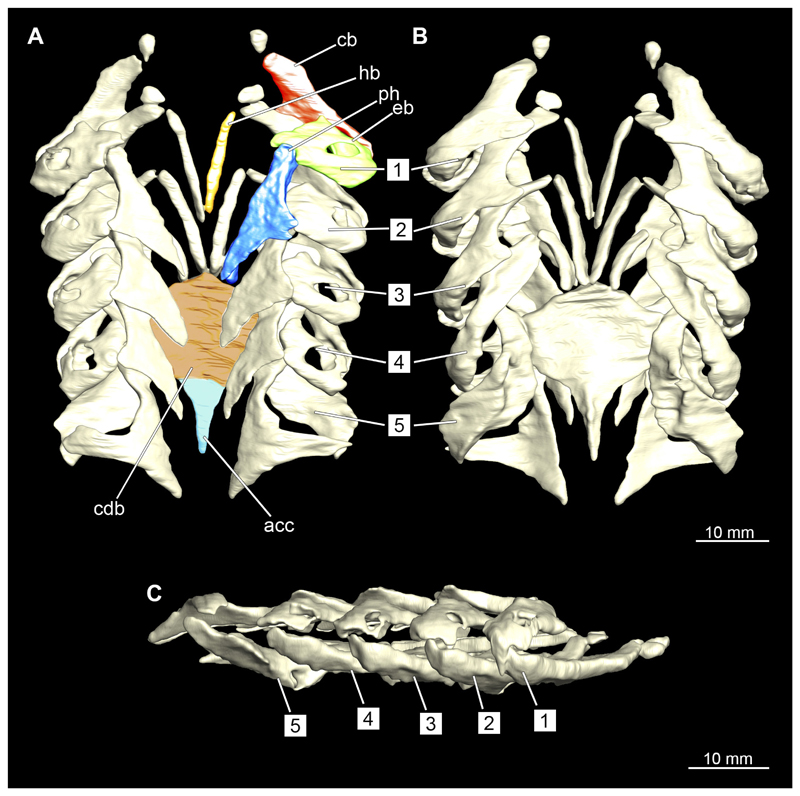
*Chiloscyllium punctatum,* female: Different views of the Visceral arches of the specimen: Abbreviations: **A** dorsal, **B** ventral and **C** lateral right side as rendered surface. Abbreviations: 1-5, visceral arches 1,2,3,4,5; acc, accessory cartilage of basibranchial; bb, basibranchiale; cb, ceratobranchiale; cdb, cardiobranchiale; eb, epibranchiale; hb, hypobranchiale; pb, pharyngobran- chiale.

**Figure 24:**
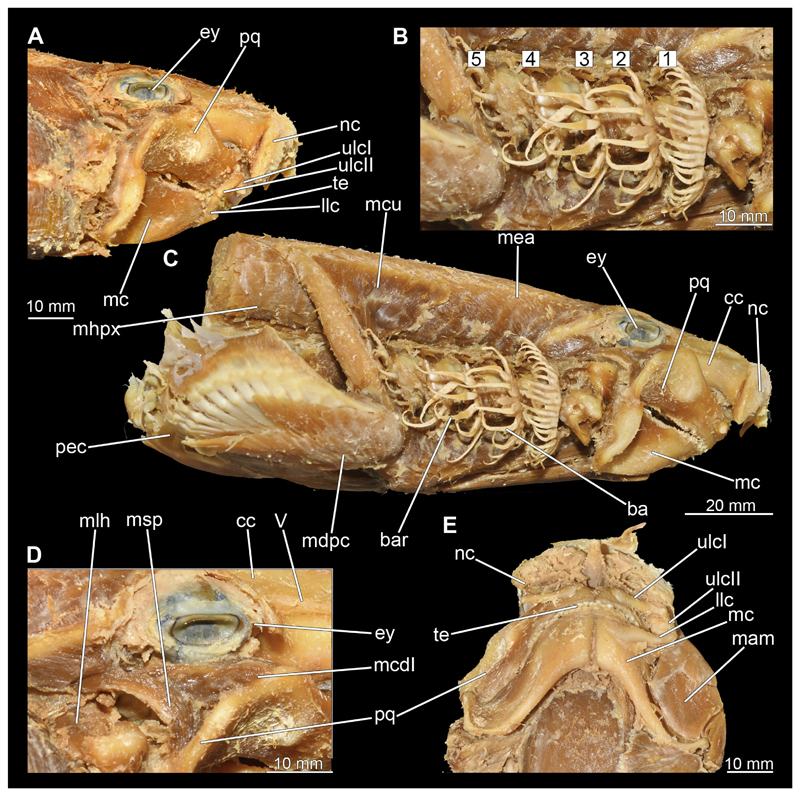
*Chiloscyllium punctatum,* female: Different views of the anterior body part of the specimen with removed epibranchial and mandibular muscles revealing the gill rays, labial cartilages and the single elements of the Musculus constrictor dorsalis. **A** ventro-lateral, **B** detail of the branchial region lateral, **C** lateral, **D** detail M. constrictor dorsalis, dorso-lateral and **E** ventral views of the dissected specimen. Abbreviations: 1–5, visceral arches 1,2,3,4,5; ba, branchial arch; bar, branchial rays; cc, chondrocranium; ey, eye; llc, lower labial cartilage; mam, musculus adductor mandibulae; mc, Meckel’s cartilage; mcdI, musculus constrictor dorsalis I; mcu, musculus cucullaris; mdpc, musculus depressor pectoralis; mea, musculus epiaxialis mhpx, musculus hypaxialis; mlh, musculus levator hyomandibulae; msp, musculus spiracularis; nc, nasal capsule; pec, pectoral fin; pq, palatoquadratum; te, teeth; ulcI, upper labial cartilage I; ulcII, upper labial cartilage II.

Posterior to the hyoid arch are the branchial arches (Figs 21, 22, 23, 24,) consisting of five pairs of arches (Shirai 1992), which is also the case in *C. punctatum.* Each individual arch comprises a sequence of characteristic single elements, i.e., the pharyngobranchialia, epibranchialia, ceratobranchialia, hypobranchialia, and basibranchialia (Figs 21A, 23A). The pharyngobranchial is the dorsal-most cartilage of the branchial arches, which is connected dorsally to the vertebral column via connective tissues. Cranially, the very flat element extends into a triangular shape. In the middle, the cartilaginous element runs in a relatively straight line postero-anteriad. Antero-laterally, the pharyngobranchial articulates with the epibranchial. The epibranchial is rather stout and has a large central foramen for the branches of the branchial nerve. The pharyngobranchial of the posterior-most two gill arches and the epibranchial of the last gill arch are fused into a single large element, the gill pickax. This is medially elongated with a spatulate extension on the outer lateral side. The ceratobranchial is elongated and has a median extension on half of its length. The distal end articulates with the epibranchial element. With the anterior end, the ceratobranchial articulates from the second to fourth arch with the hypobranchial, in arch five with the basibranchial and in arch one with the basihyal. The hypobranchial is only present in arches 2–4. This element is elongated and rather narrow. It extends roughly in a cranio-caudal direction and articulates anteriorly with the ceratobranchial and posteriorly with the basibranchial. The length of the hypobranchial decreases continuously from arch two to four.

The basibranchial generally consists of a single unpaired element. It lies medial on the ventral side of the branchial arches. This element is diamond shaped. Caudally, there is an articulation with the accessory cartilage, which is small and narrow.

The gill rays are not visible in the micro-CT scan, but are distinct in the dissection (Figs 22B–C, 24B–C). There are no significant differences in the structure or morphology of the branchial apparatus between the two sexes.

#### 3.2.7 Muscles associated with the visceral skeleton

##### 3.2.7.1 Mandibular muscles

###### 3.2.7.1.1 Musculus levator labii

**Figure 25:**
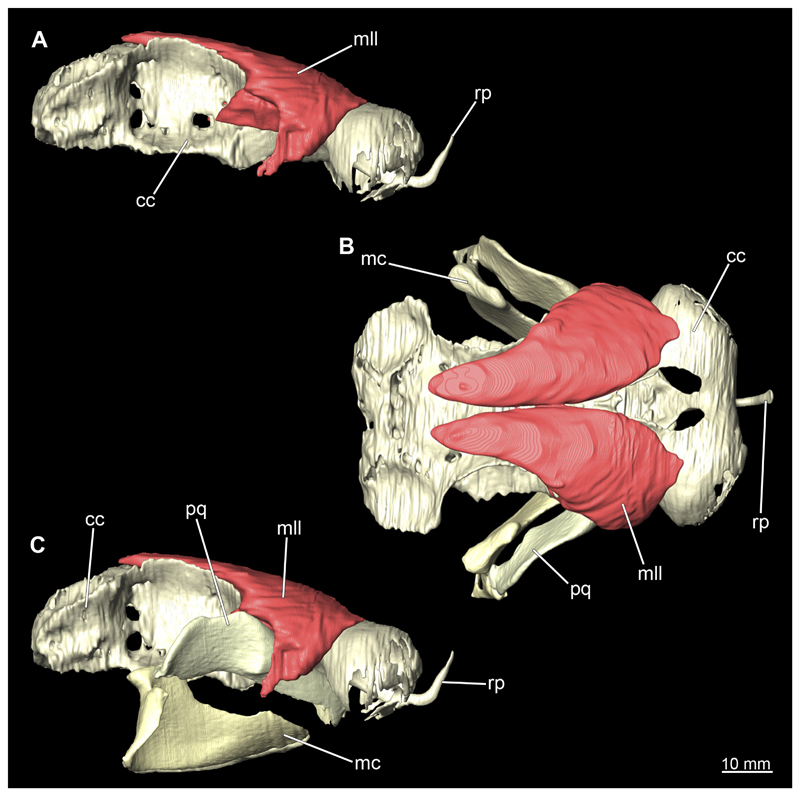
*Chiloscyllium punctatum,* male: Different views of the Musculus levator labii, together with the Chondrocranium and the mandibular arch of the specimen. **A** lateral (only with chondrocranium), **B** dorsal (with mandibular arch), and **C** lateral (with mandibular arch) views as rendered surface. Abbreviations: cc, chondrocranium; mc, Meckel’s cartilage; mll, musculus levator labii; pq, palatoquadratum; rp, rostral process.

**Figure 26:**
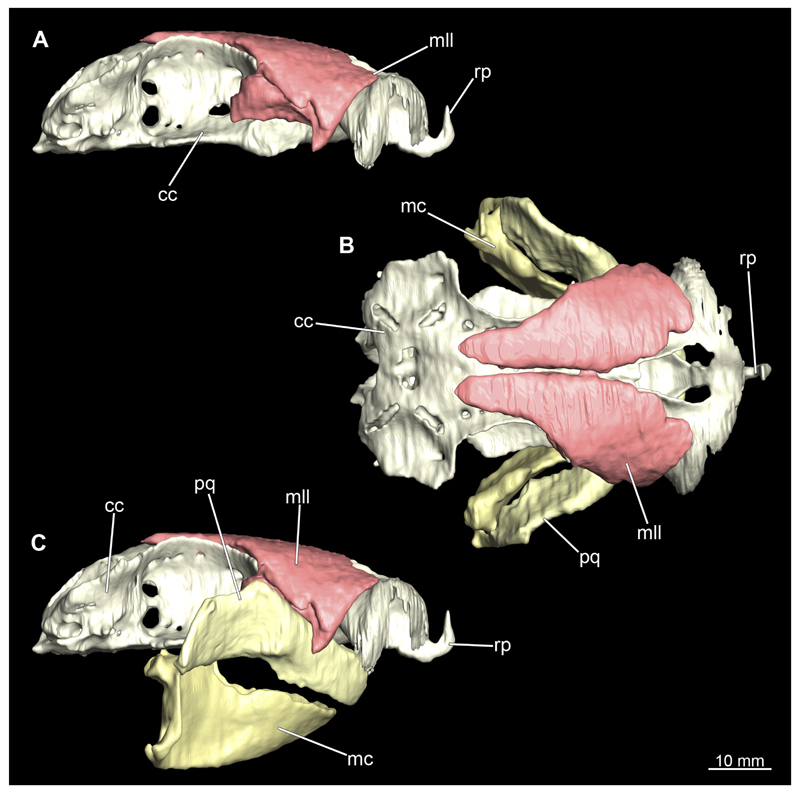
*Chiloscyllium punctatum,* female: Different views of the Musculus levator labii, together with the Chondrocranium and the mandibular arch of the specimen. **A** lateral (only with chondrocranium), **B** dorsal (with mandibular arch) and **C** lateral (with mandibular arch) views as rendered surface. Abbreviations: cc, chondrocranium; mc, Meckel’s cartilage; mll, musculus levator labii; pq, palatoquadratum; rp, rostral process.

At the posterior end of the nasal capsules, the Musculus levator labii superioris is attached dorsally to the chondrocranium (Figs 25, 26, 28A, C–D, 30,A,C). The fact that the muscle attaches directly to the neurocranium, and not through a tendon as in squaliform sharks, identifies *C. punctatum* as a representative of galeomorph sharks, a criterion used for establishing the monophyly of galeomorphs; Soares and Carvalho (2013). In dorsal view, it originates anteriorly at the same level as the subnasal fenestrae and extends dorsally to the endolymphatic and perilymphatic foramina (Figs 25B, 26B, 28A, 33A), where it meets the Musculus epaxialis without merging with it. Laterally, the Musculus levator labii reaches around the palatoquadratum both anteriorly and posteriorly, while the posterior strand inserts on the anterior wall of the orbit (Figs 25A,C, 26A,C). The anterior cord does not extend as far ventrally in the female as in the male specimen, in which it extends slightly beyond the lower edge of the palatoquadratum (Figs 25A,C).

###### 3.2.7.1.2 Musculus adductor mandibulae

**Figure 27:**
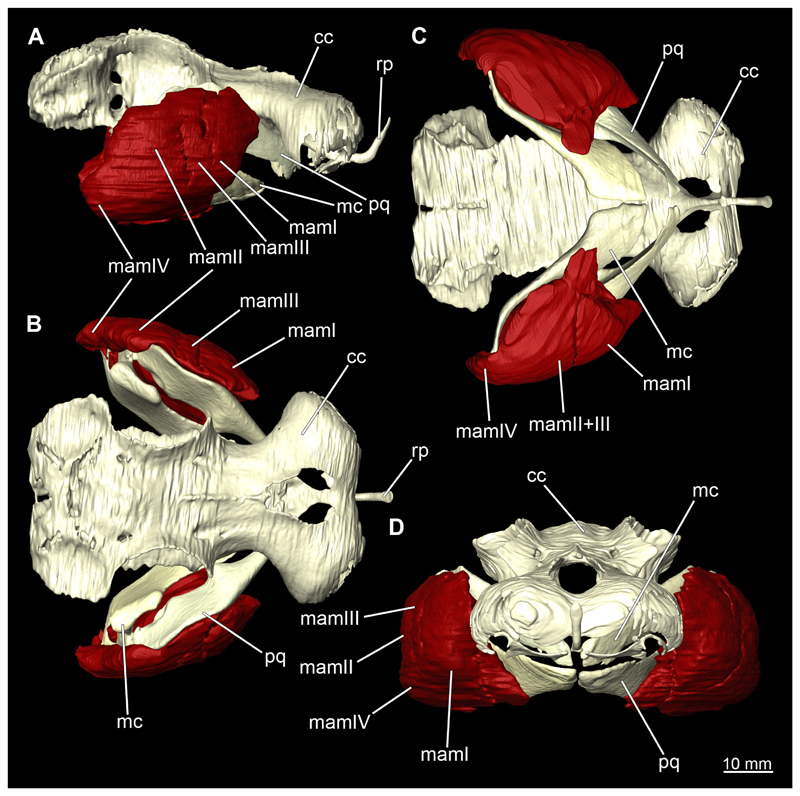
*Chiloscyllium punctatum,* male: Different views of the Musculus adductor mandibulae, together with the Chondrocranium and the mandibular arch of the specimen. **A** lateral, **B** dorsal, **C** ventral and **D** anterior views as rendered surface. Abbreviations: cc, chondrocranium; mc, Meckel’s cartilage; mam I-IV, musculus adductor mandibulae I-IV; pq, palatoquadratum; rp, rostral process.

**Figure 28:**
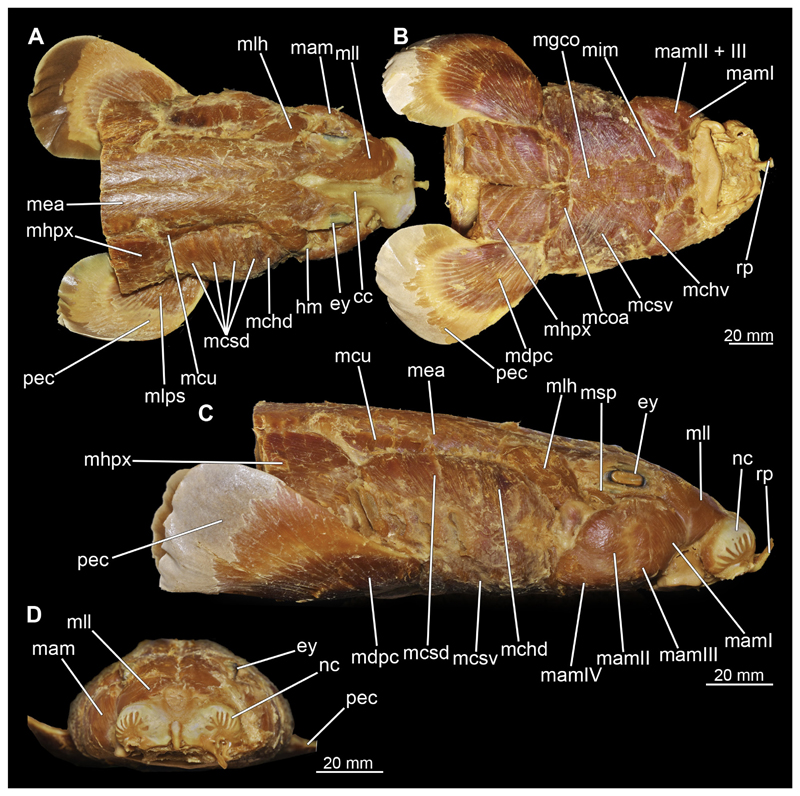
*Chiloscyllium punctatum,* male: Different perspectives of the anterior part of dissected specimen. In this dissection step the skin and the biggest part of the connective tissue were removed clearly showing large parts of the muscles of the anterior body region. **A** dorsal, **B** ventral, **C** lateral and **D** anterior views of the dissected specimen. Abbreviations: cc, chondrocranium; ey, eye; hm, hyomandibula; mam, musculus adductor mandibulae; mamI–IV, musculus adductor mandibulae I–IV; mchd, musculus constrictor hyoideus dorsalis; mchv, musculus constrictor hyoideus ventralis; mcoa, musculus coraco arcualis; mcsd, musculus constrictor superficialis dorsalis; mcsv, musculus constrictor superficialis ventralis; mcu, musculus cucullaris; mdpc, musculus depressor pectoralis; mea, musculus epiaxialis; mgco, musculus genio coracoideus; mhpx, musculus hypaxialis; mim, musculus intermandibularis; mlh, musculus levator hyomandibulae; mll, musculus levator labii; mlps, musculus levator pectoralis superficialis; msp, musculus spiracularis; nc, nasal capsules; pec, pectoral fin; rp, rostral process.

**Figure 29:**
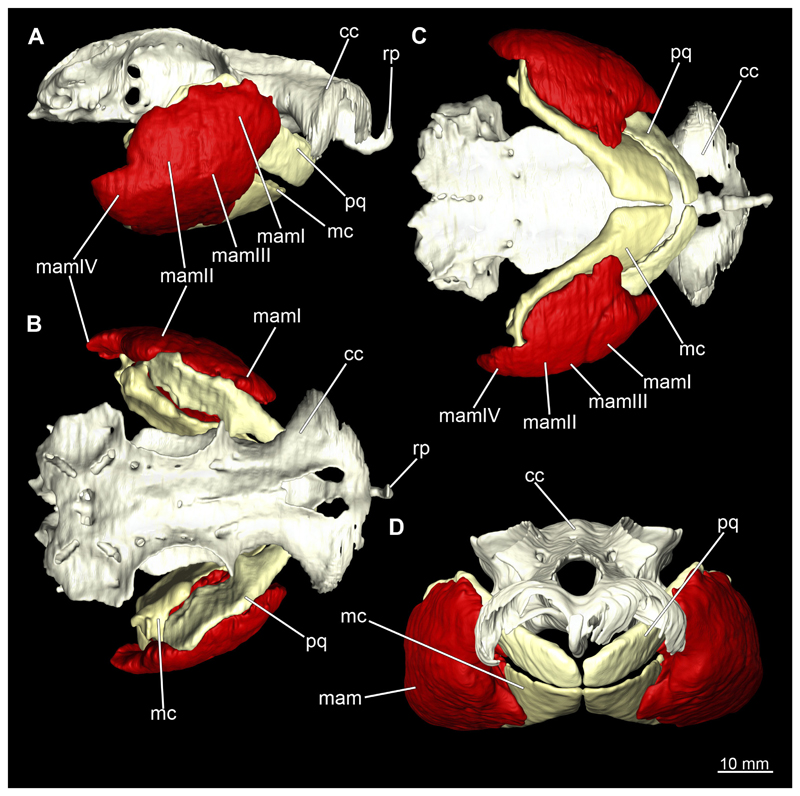
*Chiloscyllium punctatum,* female: Different views of the Musculus adductor mandibulae, together with the Chondrocranium and the mandibular arch of the specimen. **A** lateral (only with chondrocranium), **B** dorsal, **C** ventral and **D** anterior views as rendered surface. Abbreviations: cc, chondrocranium; mc, Meckel’s cartilage; mam I–IV, musculus adductor mandibulae I–IV; pq, palatoquadratum; rp, rostral process.

**Figure 30:**
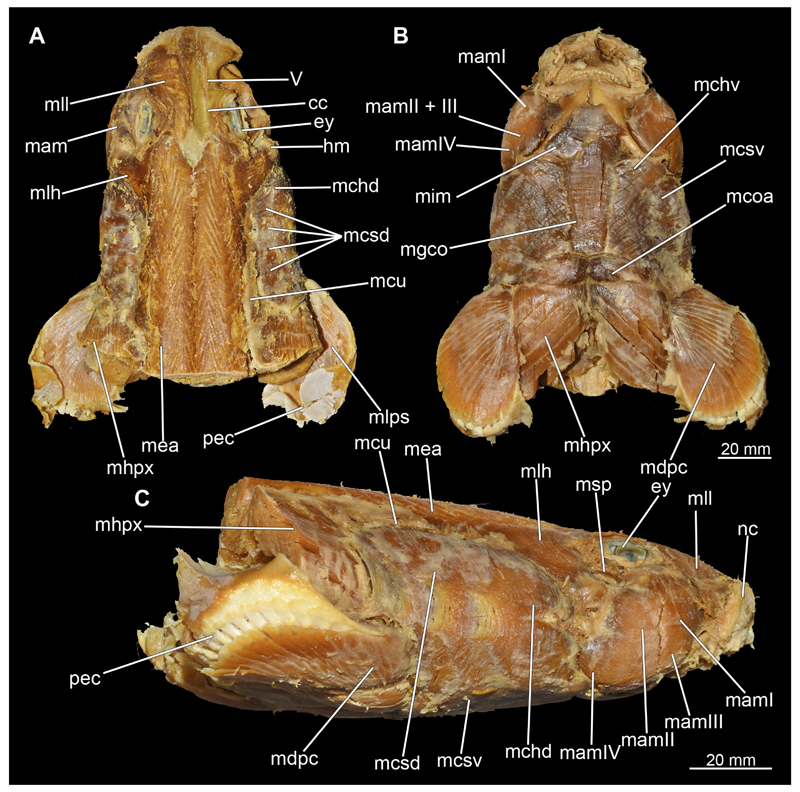
*Chiloscyllium punctatum,* female: Different perspectives of the anterior part of dissected specimen. In this dissection step the skin and the biggest part of the connective tissue were removed clearly showing large parts of the muscles of the anterior body region. **A** dorsal, **B** ventral and **C** lateral views of the dissected specimen. Abbreviations: cc, chondrocranium; ey, eye; hm, hyomandibula; mam, musculus adductor mandibulae; mamI–IV, musculus adductor mandibulae I–IV; mchd, musculus constrictor hyoideus dorsalis; mchv, musculus constrictor hyoideus ventralis; mcoa, musculus coraco arcualis; mcsd, musculus constrictor superficialis dorsalis; mcsv, musculus constrictor superficialis ventralis; mcu, musculus cucullaris; mdpc, musculus depressor pectoralis; mea, musculus epiaxialis; mgco, musculus genio coracoideus; mhpx, musculus hypaxialis; mim, musculus intermandibularis; mlh, musculus levator hyomandibulae; mll, musculus levator labii; mlps, musculus levator pectoralis superficialis; msp, musculus spiracularis; nc, nasal capsules; pec, pectoral fin; rp, rostral process; V, Nervus trigeminalis (CN V).

The Musculi adductor mandibulae are the primary jaw closing muscles in sharks. They generally consist of four subgroups labelled I to IV (Figs27, 28B–C, 29A–C, 30B–C). Although superficially separated, the internal fibres of all subgroups intersect.

The M. adductor mandibulae I is the most anterior part (Figs 27, 28B–C, 29A–C, 30B–C). Its fibres have a dorso-ventral orientation. It is posteriorly positioned to the M. adductor mandibulae II in the dorsal region and to the M. adductor mandibulae III in the ventral region (Figs 27, 28B–C, 29A–C, 30B–C). There is a blending of the fibres at the ventral border of M. adductor mandibulae I and III. The M. adductor mandibulea II extends diagonally at 45° from antero-dorsal to postero-ventral. Typically for *Chiloscyllium,* this muscle strand is situated dorsal to the M. adductor mandibulae III (Soares and Carvalho 2013). It inserts at the palatoquadratum and extends towards the Meckel’s cartilage where only a small part of the muscle is attached to it. The M. adductor mandibulae III, on the other hand, is connected to both elements of the mandibular arch to approximately the same extent. The postero-ventral portion of the mandibular muscle is the M. adductor mandibulae IV. This is fused with the upper and lower jaws and covers the area of the quadrato-mandibular joint. In both sexes the jaw muscles are developed to the same extent and do not differ in direction or arrangement.

##### 3.2.7.2 Hyoidal muscles

###### 3.2.7.2.1 Musculus intermandibularis

**Figure 31:**
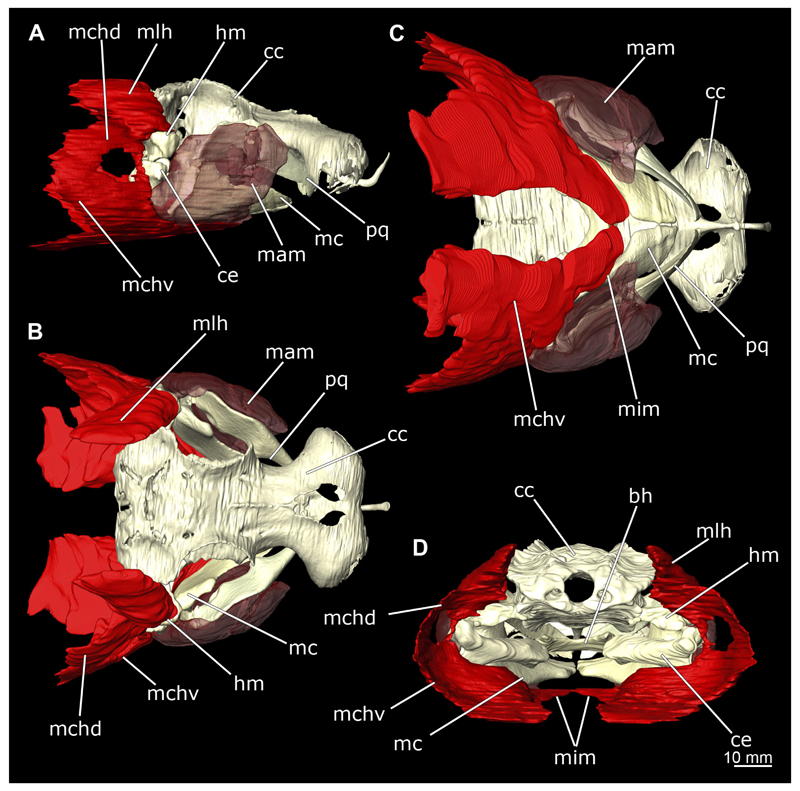
*Chiloscyllium punctatum,* male: Different views of the postcranial muscles, together with the Chondrocranium and the mandibular and hyoid arch of the specimen. **A** lateral, **B** dorsal, **C** ventral and **D** posterior views as rendered surface. Abbreviations: bh, basihyale; cc, chondrocranium; ce, ceratohyal; hm, hyomandibula; mam, musculus adductor mandibulae; mc, Meckel’s cartilage; mchd, musculus constrictor hyoideus dorsalis; mchv, musculus constrictor hyoideus ventralis; mim, musculus intermandibularis; mlh, musculus levator hyomandibulae; pq, palatoquadratum.

The Musculus intermandibularis is a paired, very slender muscle whose fibres run transversely to the longitudinal axis of the body. Both muscles attach to almost the entire length of the ventral edge of the Meckel’s cartilage from the distal end of the Meckel’s cartilage, at the level of the sustentaculum, almost to the symphysis (Figs 28B, 30B, 31C–D). This is where both parts of the muscle join. Typically for *Chiloscyllium,* this muscle is strongly connected to the Musculus constrictor hyoideus ventralis. Since the muscle forms a very slender and thin muscle bundle, it is hardly visible in the CT-Images. It could only be reconstructed in the male, but it is clearly visible in both sexes in the dissection (Figs 28B, 30B).

###### 3.2.7.2.2 Musculus constrictor dorsalis

**Figure 33:**
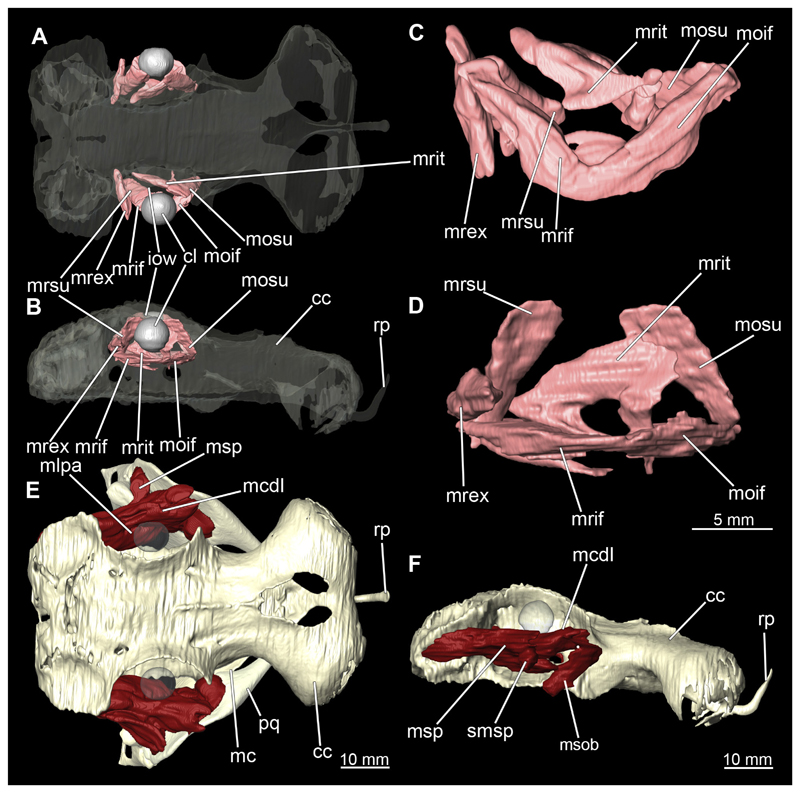
*Chiloscyllium punctatum*, male: Different views of the extraocular muscles and the Musculus constrictor dorsalis together with the lens, the Chondrocranium, the mandibular arch and of the specimen. **A** dorsal, **B** lateral, **C** left extraocular muscles ventral, **D** right extraocular muscles lateral, **E** Musculus constrictor dorsalis with chondrocranium and mandibular arch dorsal and **F** Musculus constrictor dorsalis with chondrocranium and mandibular arch lateral views as rendered surface. Abbreviations: cc, chondrocranium; cl, crystal lense; iow, infraorbital wall; mcdI, musculus constrictor dorsalis I; mlpa, musculus levator palatoquadrati; moif, musculus obliquus superioris mosu, musculus suborbitalis; mrex, musculus rectus externus; mrif, musculus rectus inferioris; mrsu, musculus rectus superioris; msob, musculus suborbitalis; msp, musculus spiracularis; pq, palatoquadratum; rp, rostral process; smsp, subdivision of musculus spiracularis.

**Figure 34:**
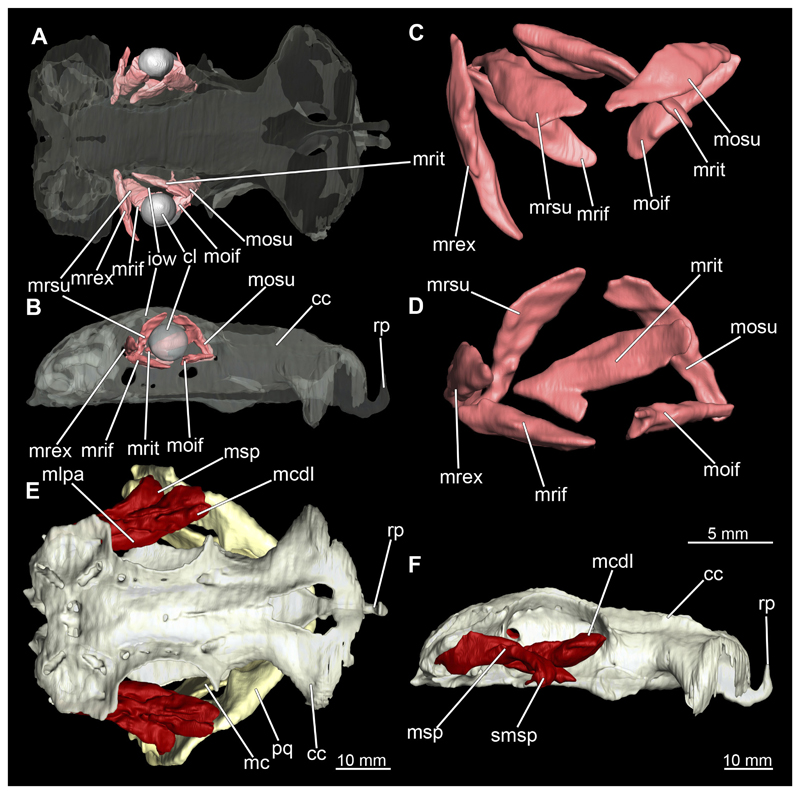
*Chiloscyllium punctatum*, female: Different views of the extraocular muscles and the Musculus constrictor dorsalis together with the lens, the Chondrocranium, the mandibular arch and of the specimen. **A** dorsal, **B** lateral, **C** left extraocular muscles ventral, **D** right extraocular muscles lateral, **E** Musculus constrictor dorsalis with chondrocranium and mandibular arch dorsal and **F** Musculus constrictor dorsalis with chondrocranium and mandibular arch lateral views as rendered surface. Abbreviations: cc, chondrocranium; cl, crystal lense; iow, infraorbital wall; mcdI, musculus constrictor dorsalis I; mlpa, musculus levator palatoquadrati; moif, musculus obliquus superioris mosu, musculus suborbitalis; mrex, musculus rectus externus; mrif, musculus rectus inferioris; mrsu, musculus rectus superioris; msp, musculus spiracularis; pq, palatoquadratum; rp, rostral process; smsp, subdivision of musculus spiracularis.

The Musculus constrictor dorsalis runs along the lower edge of the orbit, which appears to split into several individual bundles anteriorly (Figs 33E–F, 34E–F, 22D, 24D). It attaches to the ventral side of the postorbital process and is slightly antero-ventrally oriented. The Musculus levator palatoquadrati represents the innermost part of the M. constrictor dorsalis (Figs 33E and 34E). It inserts postero-ventral on the postorbital process and anteriorly slightly below the Ligamentum mandibulo-palatoquadrati. The constrictor dorsalis I muscle, which attaches centrally to the dorsal edge of the palatoquadrate, runs further distally parallel to the Musculus levator palatoquadrati. It is slightly longer than the M. levator palatoquadrati (Figs 3E–F, 34E–F, 22D, 24D). In *Chiloscyllium* and *Hemiscyllium* the muscle is divided into two individual bundles according to Goto (2001), one with a proximal and the other with a distal tendon. The bipartition is clearly visible in both sexes in the micro-CT models (Figs 33E and 34E). The most distal of the three muscles is the Musculus spiracularis (Figs 33E–F, 34E–F, 22D, 24D). It originates near the hyomandibular fossa and inserts on the distal end of the palatoquadratum. Typical for *Chiloscyllium* is the small subdivision of the spiracularis, which branches off at about two-thirds of its length (Figs 33F and 34F). This strand inserts on the mandibular knob of the lower jaw. The Musculus constrictor dorsalis forms the anterior wall of the spiracle in *Chiloscyllium.* In the male specimen, parts of the suborbitalis muscle were reconstructed.

###### 3.2.7.2.3 Musculus constrictor hyoideus

This muscle is divided into dorsal and ventral parts. The dorsal part in turn is divided into the Musculus levator hyomandibulae and Musculus constrictor hyoideus dorsalis (Figs 27 and 29). The former inserts in the otic region on the chondrocranium and extends lateroventrally to the hyomandibula, to which it is firmly attached. Dorsally, the levator hyomandibulae muscle borders the M. epaxialis. The Musculus constrictor hyoideus dorsalis arises at the lateral edge of the M. epaxialis and extends ventrally (Figs. 27A–B and 29A–B).

The paired Musculus constrictor hyoideus ventralis has transversal fibres. Ventrally, the muscle attaches to the distal end of the Meckel’s cartilage (Figs. 27A,C and 29A,C). According to Soares and Carvalho (2013), a thin cord attaches superficially to the distal tip of the ceratohyal. However, this was neither observed in the 3D models nor in the dissected specimens. Posteriorly, the M. constrictor hyoideus ventralis is connected to the M. constrictor hyoideus dorsalis in the branchial region by interconnecting muscle fibres.

##### 3.2.7.3 Hypobranchial muscles

**Figure 35:**
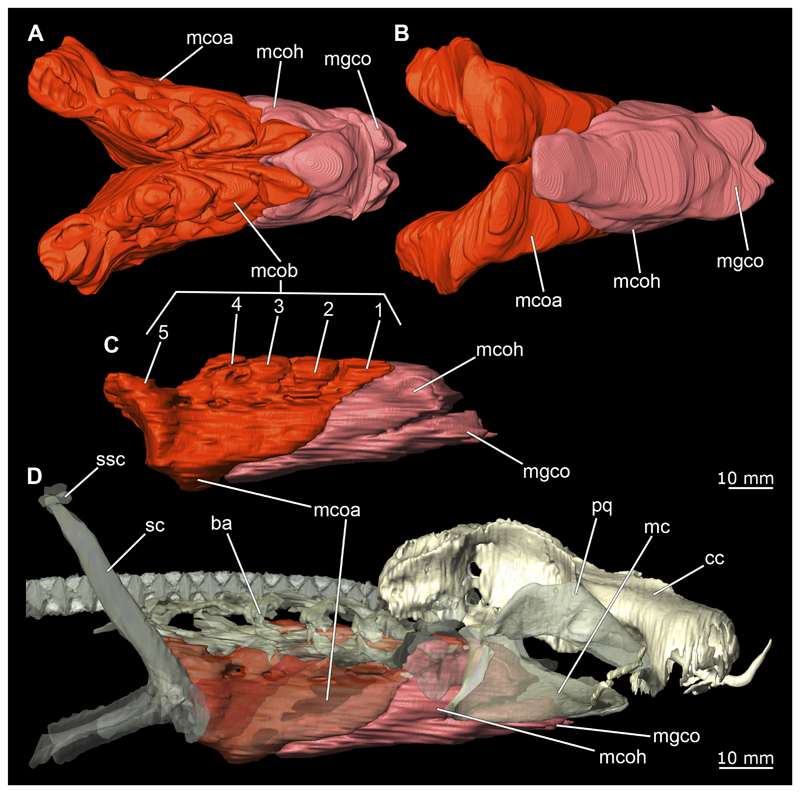
*Chiloscyllium punctatum*, male: Overview of the individual components of the hypobranchial muscles together with the interacting elements of the feeding apparatus. **A** hypobranchial muscles dorsal, **B** hypobranchial muscles ventral, **C** hypobranchial muscles lateral, **D** hypobranchial muscles with mandibular, hyoid and visceral arches lateral, **E** hypobranchial muscles with visceral arches lateral, **F** hypobranchial muscles with basihyale and parts of visceral arches as rendered surface. Abbreviations: cc, chondrocranium; mc, Meckel’s cartilage; mcoa, musculus coraco-arcualis; mcob (1–5), musculus coraco-branchialis; mcoh, musculus coraco-hyoideus; mgco, musculus genio-coracoideus; pq, palatoquadratum; sc, scapula; ssc, suprascapula.

**Figure 36:**
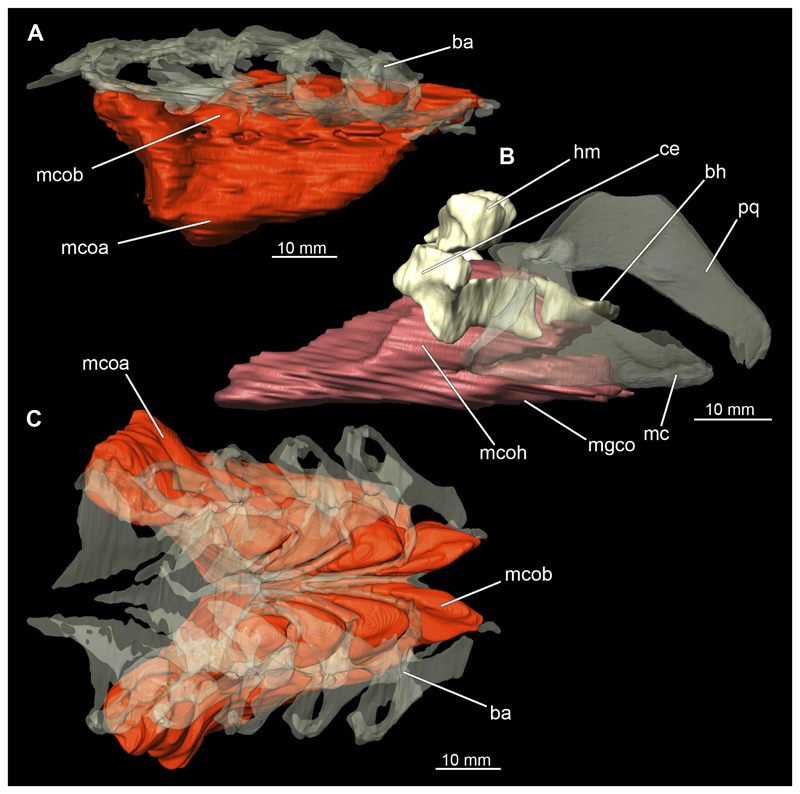
*Chiloscyllium punctatum*, male: Hypobranchial muscles together with the interacting elements hyoid and visceral arches. **A** musculus coraco-arcualis and musculus coraco-branchialis with visceral arches lateral, **B** musculus coraco-hyoideus and musculus genio-coracoideus with hyoid arch and mandibular arch lateral and **C** musculus coraco-branchialis with branchial arches dorsal as rendered surface. Abbreviations: ba, branchial arches, bh, basihyale; ce, ceratohyal; hm, hyomandibula; mc, Meckel’s cartilage; mcoa, musculus coraco-arcualis; mcob, musculus coraco-branchialis; mcoh, musculus coraco-hyoideus; mgco, musculus genio-coracoideus; pq, palatoquadratum.

**Figure 37:**
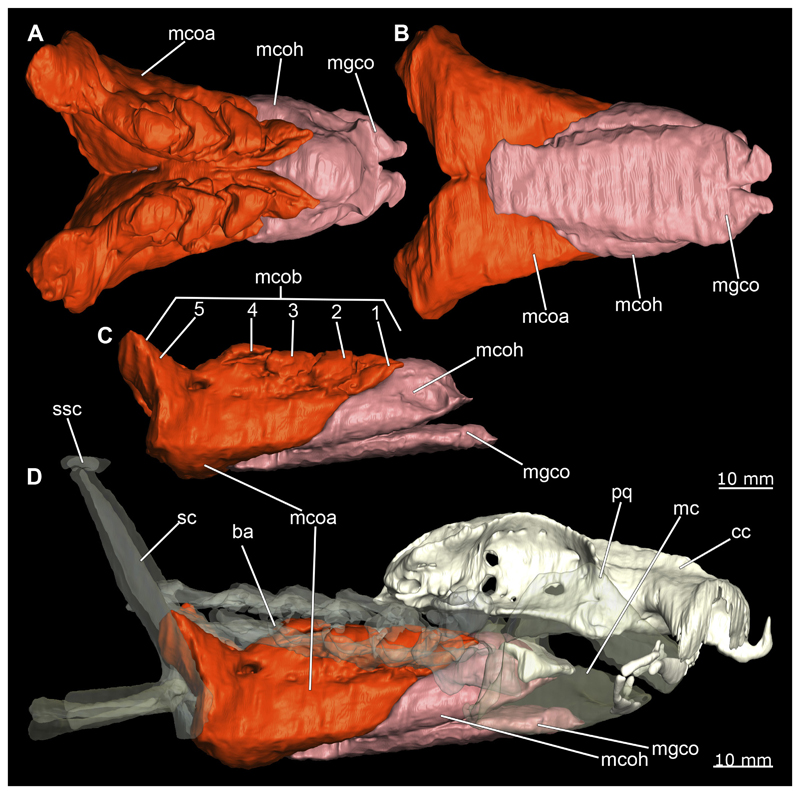
*Chiloscyllium punctatum*, female: Overview of the individual components of the hypobranchial muscles together with the interacting elements of the feeding apparatus. **A** hypobranchial muscles dorsal, **B** hypobranchial muscles ventral, **C** hypobranchial muscles lateral, **D** hypobranchial muscles with mandibular, hyoid and visceral arches lateral, **E** hypobranchial muscles with visceral arches lateral, **F** hypobranchial muscles with basihyale and parts of visceral arches. Abbreviations: cc, chondrocranium; mc, Meckel’s cartilage; mcoa, musculus coraco-arcualis; mcob (1–5), musculus coraco-branchialis; mcoh, musculus coraco-hyoideus; mgco, musculus genio-coracoideus; pq, palatoquadratum; sc, scapula; ssc, suprascapula.

**Figure 38:**
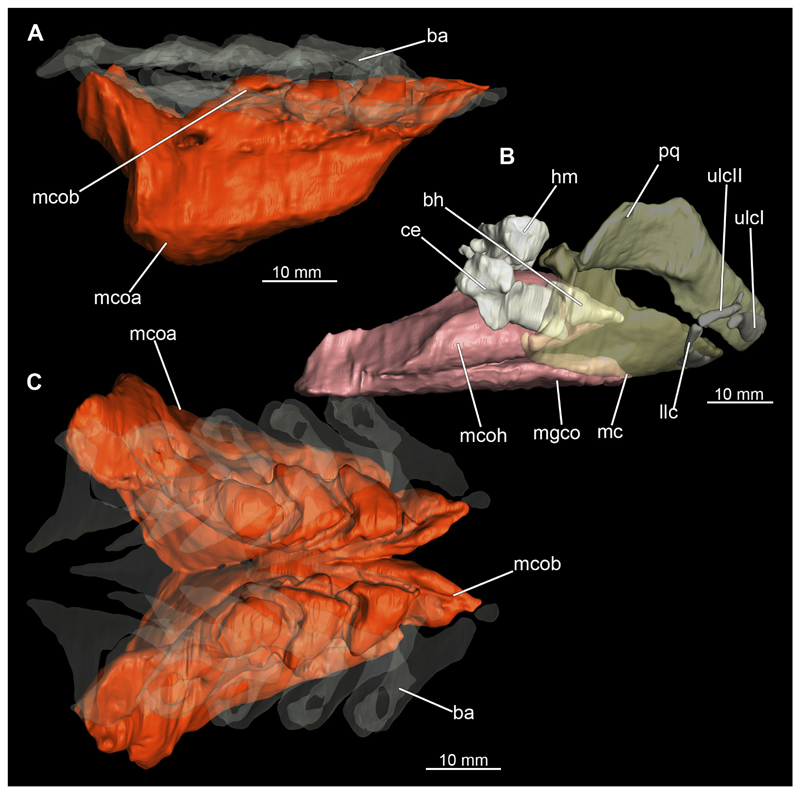
*Chiloscyllium punctatum*, female: Hypobranchial muscles together with the interacting elements hyoid and visceral arches. **A** musculus coraco-arcualis and musculus coraco-branchialis with visceral arches lateral, **B** musculus coraco-hyoideus and musculus genio-coracoideus with hyoid arch and mandibular arch lateral and **C** musculus coraco-branchialis with branchial arches dorsal as rendered surface. Abbreviations: ba, branchial arches, bh, basihyale; ce, ceratohyal; hm, hyomandibula; llc, lower labial cartilage; mc, Meckel’s cartilage; mcoa, musculus coraco-arcualis; mcob, musculus coraco-branchialis; mcoh, musculus coraco-hyoideus; mgco, musculus genio-coracoideus; pq, palatoquadratum; ulcI, upper labial cartilage I; ulcII, upper labial cartilage II.

In Orectolobiformes, the hypobranchial musculature typically consists of the Musculus genio-coracoideus, the Musculus rectus-cervicis and the Musculus coraco-branchialis (Goto 2001). The M. genio-coracoideus extends from a fascia of the M. rectus cervicis ventrally to the symphysis of the lower jaw (Figs. 28B, 30B, 35, 37). There it inserts on the ventral surface of the Meckel’s cartilage. It is relatively narrow, long and tapers both anteriorly and posteriorly into a rounded end (Figs. 35B and 37B). The M. rectus-cervicis consists of the M. coraco-arcualis and the M. coraco-hyoideus, which are both clearly separated by a septum (Figs. 35A–C and 37A–C). The M. rectus-cervicis runs parallel dorsal to the M. genio-coracoideus. It consists of a pair of parallel muscle bundles. On about one third of the anterior part of the M. rectus-cervicis, the muscle bundles are separated from the posterior part by a septum. The anterior part, called the M. coraco-hyoideus, attaches to the ventral side of the basihyale (Figs. 36B and 38B). The posterior part, the M. coraco-arcualis, originates on the ventral side of the coracoid and further dorsally on the anterior tip of the Muscoli hypaxialis (Figs. 35D and 37D).

The M. coraco-hyoideus and the M. genio-coracoideus originate from a fascia of the M. rectus-cervicis. The M. coraco-hyoideus inserts anteriorly on the caudal end and the ventral surface of the basihyal. The five Muscoli coraco-branchialis posteriorly or dorsally of the M. rectus-cervicis vary only slightly in size (Figs. 35C, 36A,C, 37C, 38A,C). The posteriormost strand appears clearly separated from the four anterior ones (Figs. 35C, 36A, 37C, 38A). It points caudally in a semi-arch. The four posterior strands of the M. coraco-branchialis originate in the anterior part of the coracoid and attach to the Muscoli hypobranchialis and Muscoli ceratobranchialis (Shirai, 1992).

#### 3.2.8 Eyes

In Chiloscyllium punctatum the eyes are relatively small. They are located slightly posterior to the mouth opening and slightly anterior to the spiracles (Figs. 1A–B and 2A–B). The pupils are more or less slit-shaped, depending on the amount of incoming radiation, and slope from posterior to anterior at an angle of 45°. In the micro-CT images, the lens is particularly bright, as it seems to have absorbed a considerable amount of iodine. Around the eyeball, a series of narrow muscle bands are visible representing the oculomotor muscles.

In Chiloscyllium punctatum there are six oculomotor muscles, two pairs of muscles and two single muscles (Figs. 33A–D and 34A–D). Anteriorly, there is the Musculus obliquus superioris and the Musculus obliquus inferioris (Figs. 33A–D and 34A–D). Both are clearly separated from each other, and both originate on the anterior part of the orbit at the interorbital wall. The main function of the obliquus superioris muscle is to roll the eye anteriorly, whereas the obliquus inferioris muscle is mainly responsible for rolling the eye caudally (Goto 2001). Further posteriorly, the Musculus rectus internus is located centrally between the other five oculomotor muscles.

It originates further posteriorly on the interorbital wall, close to but clearly separated from the other three rectus muscles. At its base, there is an extra muscle bundle, which originates ventrally and joins the extra bundle of the inferior rectus muscle (not visible in the micro-CT scans) at the same point, the ocular pedicle (Goto 2001). The main bundle runs almost horizontal in an anterior direction and attaches to the eye between the two oblique muscles. It is significantly involved in adduction, i.e., the anterior movement of the eye.

As is typical of Orectolobiformes, the rectus muscles originate individually on the interorbital wall (Goto 2001) (Figs. 33A and 34A). The Musculus rectus superioris and the Musculus rectus inferioris arise as a pair between the M. rectus externus and M. rectus internus muscles. The M. rectus superioris attaches dorsally, the rectus inferioris ventrally to the posterior third of the eye (Figs. 33D and 34D).

Also typical for Orectolobiformes is the additional muscle bundle on the M. rectus inferioris (not visible in micro-CT models). It arises from the interorbital wall at the base of the inferior rectus. As previously mentioned, this strand joins the extra bundle of the internal rectus at the disc of the eyestalk (Goto 2001). The main function of the superior rectus is to elevate the eye. The M. rectus inferioris, on the other hand, is mainly responsible for lowering (depressing) the eyeball (Goto 2001).

The Musculus rectus externus attaches most posteriorly and, typically for *Chiloscyllium* and *Hemiscyllium,* arises from its own fossa posterior to the foramen for the superior branch of cranial nerve V (Figs. 33A,C and 34A,C). Its main purpose is to move the eye backwards (Goto 2001).

As these are very fine muscle bundles, an exact reconstruction of the individual muscles is not easy given the resolution of the micro-CT scans. Some components, such as the extra bundles of the rectus inferioris and parts of the extra bundles of the M. rectii internus, are not visible in the reconstructions. A probable artefact, which could also be caused by the resolution, is the fusion of the M. rectus inferioris and the M. obliquus inferioris and the clearly shortened M. rectus externus in the male specimen.

#### 3.2.9 Pectoral girdle

**Figure 39:**
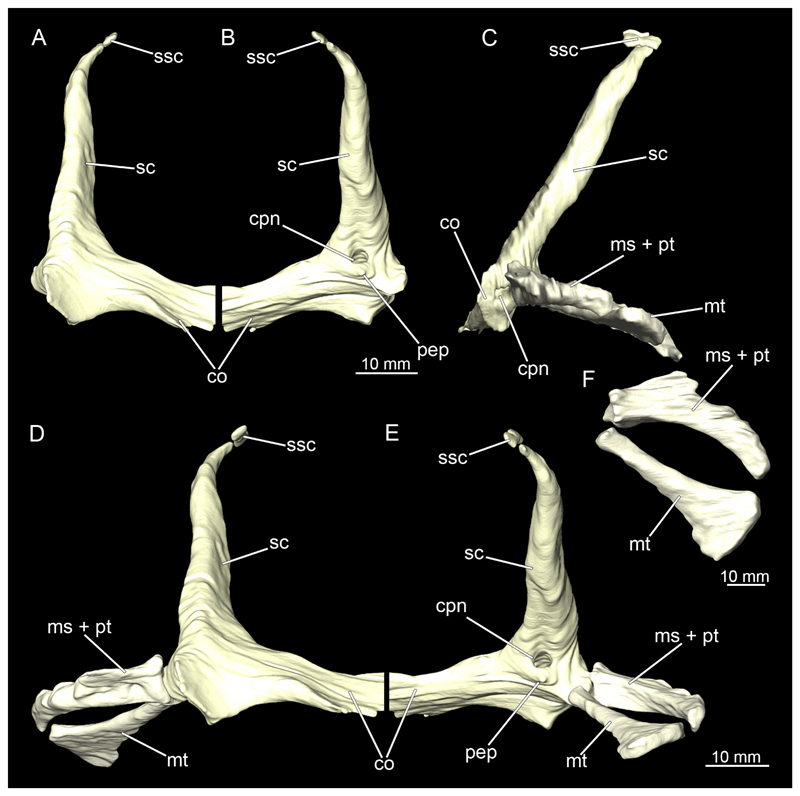
*Chiloscyllium punctatum*, male: Elements of the pectoral girdle of the specimen. **A** anterior, **B** posterior, **C** lateral with pectoral fin, **D** anterior with pectoral fin, **E** posterior with pectoral fin and **F** basal cartilages of the right pectoral fin dorsal views as a rendered surface. Abbreviations: co, coracoid; cpn, canal for pectoral nerves and brachial artery; ms, mesopterygium; mt, metopterygium; pep, process for levator pectoralis; pt, pteropterygium; sc, scapula; ssc, suprascapula.

**Figure 40:**
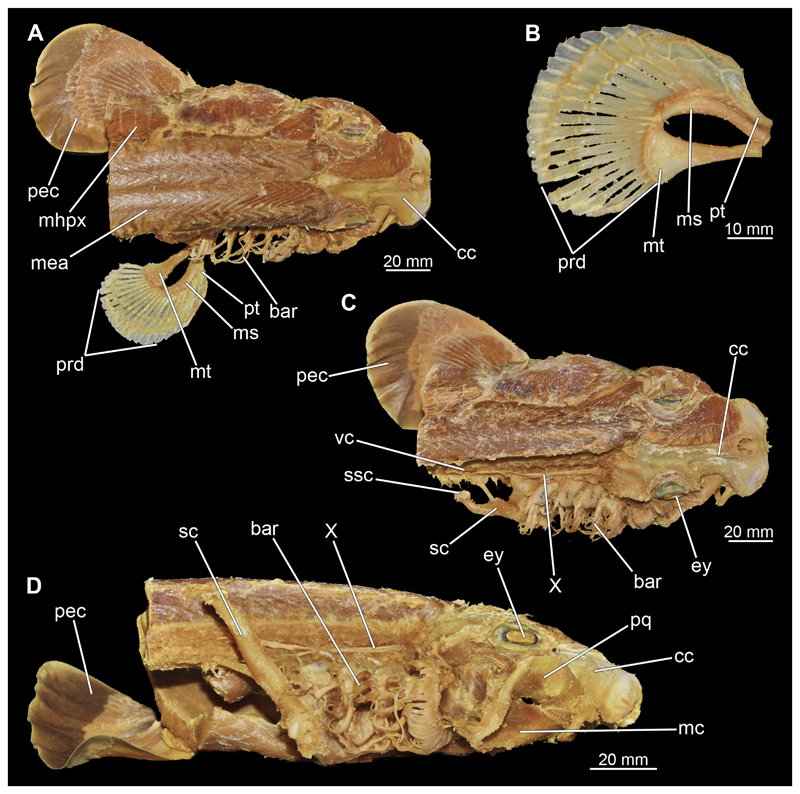
*Chiloscyllium punctatum*, male: Different perspectives of the anterior part of dissected specimen showing especially the elements of the pectoral girdle. **A** dorsal, **B** right pectoral fin ventral, **C** dorsal with removed Musculus epaxialis and removed pectoral fin and **D** lateral with removed Musculus epaxialis and removed pectoral fin; views. Abbreviations: bar, branchial rays; cc, chondrocranium; ey, eye; mc, Meckel’s cartilage; mea, musculus epaxialis; mhpx, musculus hxpaxialis; ms, mesopterygium; mt, metopterygium; pec, pectoral fin; pq, palatoquadratum; prd, radialia of the pectoral fin; pt, pteropterygium; sc, scapula; ssc, suprascapula, vc, vertebral column; X, Nervus vagus (CN X).

**Figure 41:**
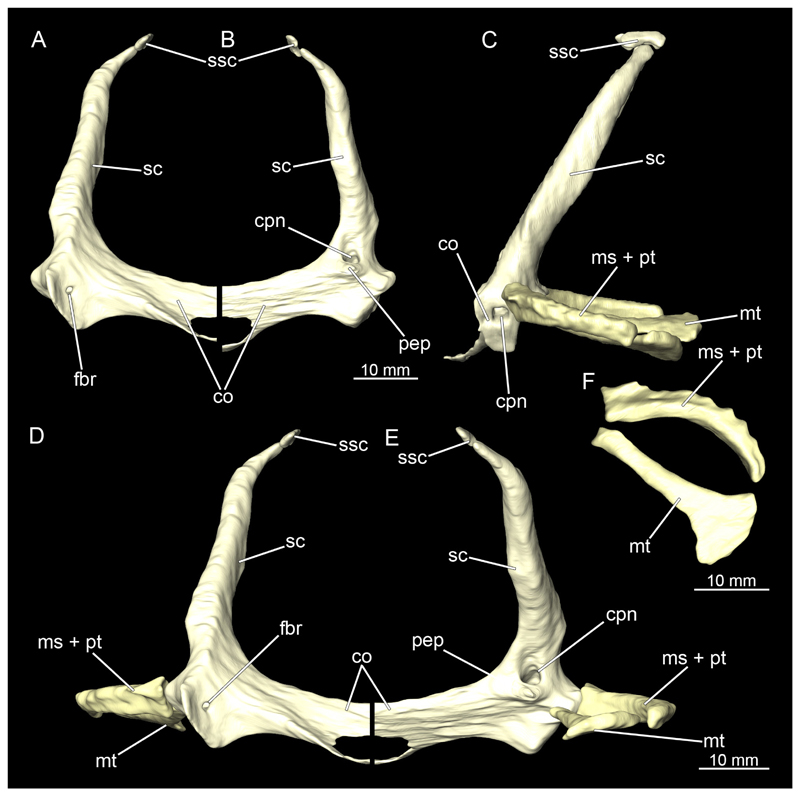
*Chiloscyllium punctatum*, female: Elements of the pectoral girdle of the specimen. **A** anterior, **B** posterior, **C** lateral with pectoral fin, **D** anterior with pectoral fin, **E** posterior with pectoral fin and **F** basal cartilages of the right pectoral fin dorsal views as a rendered surface. Abbreviations: co, coracoid; cpn, canal for pectoral nerves and brachial artery; ms, mesopterygium; mt, metopterygium; pep, process for levator pectoralis; pt, pteropterygium; sc, scapula; ssc, suprascapula.

**Figure 42:**
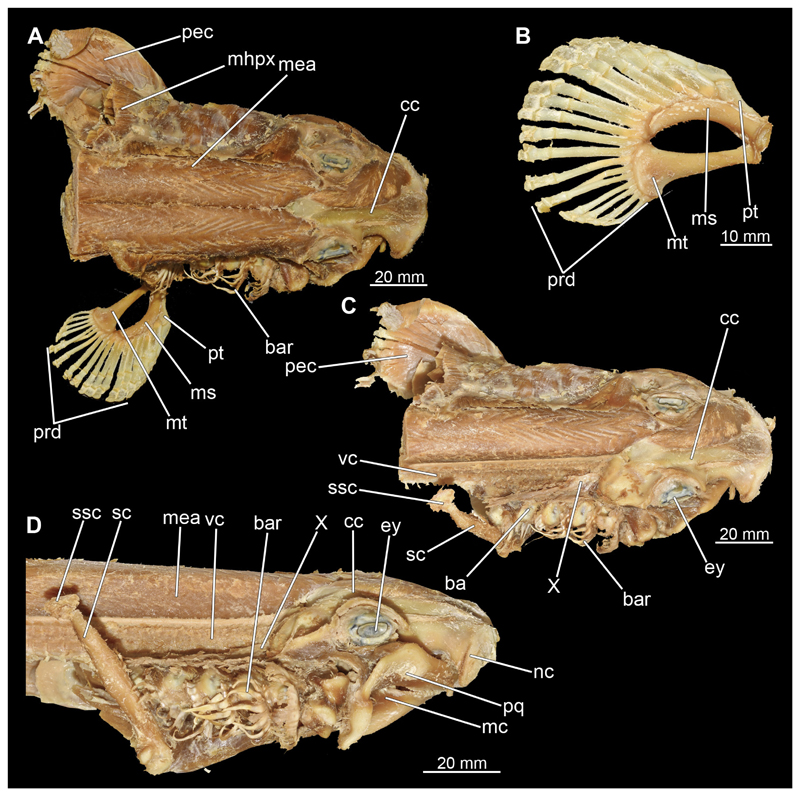
*Chiloscyllium punctatum*, female: Different perspectives of the anterior part of dissected specimen showing especially the elements of the pectoral girdle. **A** dorsal, **B** right pectoral fin ventral, **C** dorsal with removed Musculus epaxialis and removed pectoral fin and **D** lateral with removed Musculus epaxialis and removed pectoral fin; views. Abbreviations: bar, branchial rays; cc, chondrocranium; ey, eye; mc, Meckel’s cartilage; mea, musculus epaxialis; mhpx, musculus hxpaxialis; ms, mesopterygium; mt, metopterygium; nc, nasal capsules; pec, pectoral fin; pq, palatoquadratum; prd, radialia of the pectoral fin; pt, pteropterygium; sc, scapula; ssc, suprascapula, vc, vertebral column; X, Nervus vagus (CN X).

The shoulder girdle is U-shaped, which is typical for Orectolobiformes (Figs. 39A–B,D–E, 41A–B,D–E). Several individual cartilaginous elements are seamlessly fused. These are the scapula and the coracoid. The scapula is a cylindrical, vertical element that is slightly tilted caudally in lateral view (Figs. 39C, 40C, 41C, 42C–D). Dorsally on the scapula is the rudimentary suprascapula, which is very small and oval, and which is connected to the scapula by soft tissue (Figs. 39A–E, 40D, 41A–E, 42D). A suprascapula only is present in the genera *Chiloscyllium, Hemiscyllium, Ginglymostoma, Stegostoma* and *Rhincodon* within Orectolobiformes (Goto 2001). Ventrally, the coracoid is adjacent to the scapula. The coracoid appears flat and runs horizontally (Figs. 39A–B,D–E and 41A–B,D–E). The median area where the coracoids meet is widened forming a trough-like basin representing the so-called apron. This apron supports the ventral wall of the pericardial cavity. The process for the Musculus levator pectoralis is located posteriorly below the foramen for the pectoral nerves and artery (Figs. 39B,E and 41B,E). The foramen for the brachial artery penetrates the fossa for the pectoral depressor muscle longitudinally, which is typical for *Chiloscyllium* and *Hemiscyllium* within Orectolobiformes (Goto 2001).

**Figure 43:**
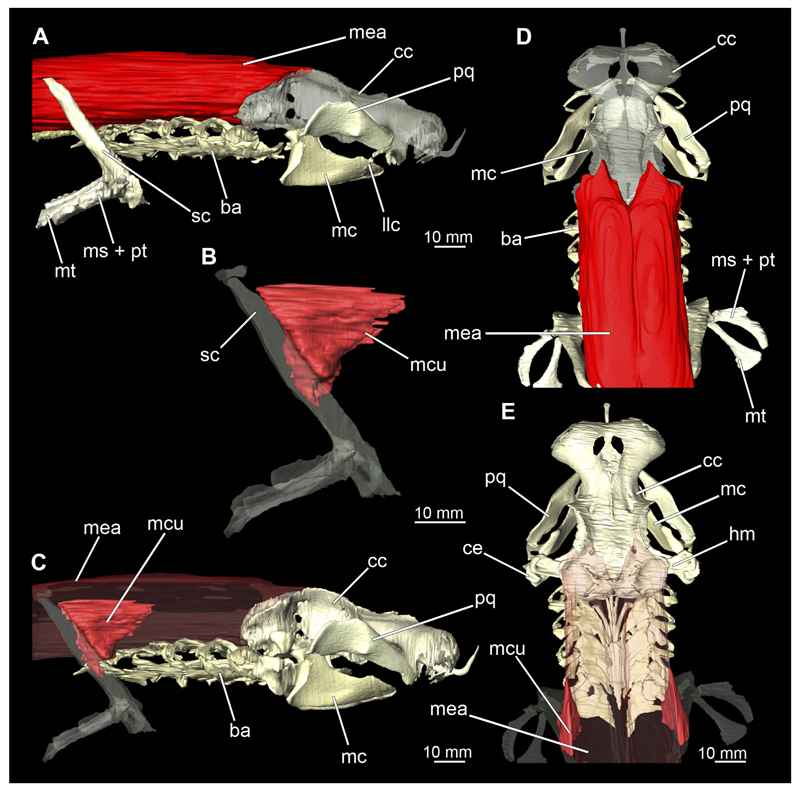
*Chiloscyllium punctatum*, male: Musculus epaxialis and Musculus cucullaris with pectoral girdle, visceral, hyoid and mandibular arches, and chondrocranium. **A** epaxialis lateral, **B** detail pectoral girdle with M. cucullaris lateral, **C** M. cucullaris lateral, **D** M. epaxialis dorsal and **E** M. cucullaris dorsal view as a rendered surface. Abbreviations: ba, branchial arches; cc, chondrocranium; ce, ceratohyal; hm, hyomandibula; llc, lower labial cartilage; mc, Meckel’s cartilage; mcu, musculus cucullaris; mea, musculus epaxialis; ms, mesopterygium; mt, metopterygium; pq, palatoquadratum; pt, pteropterygium; sc, scapula.

**Figure 44:**
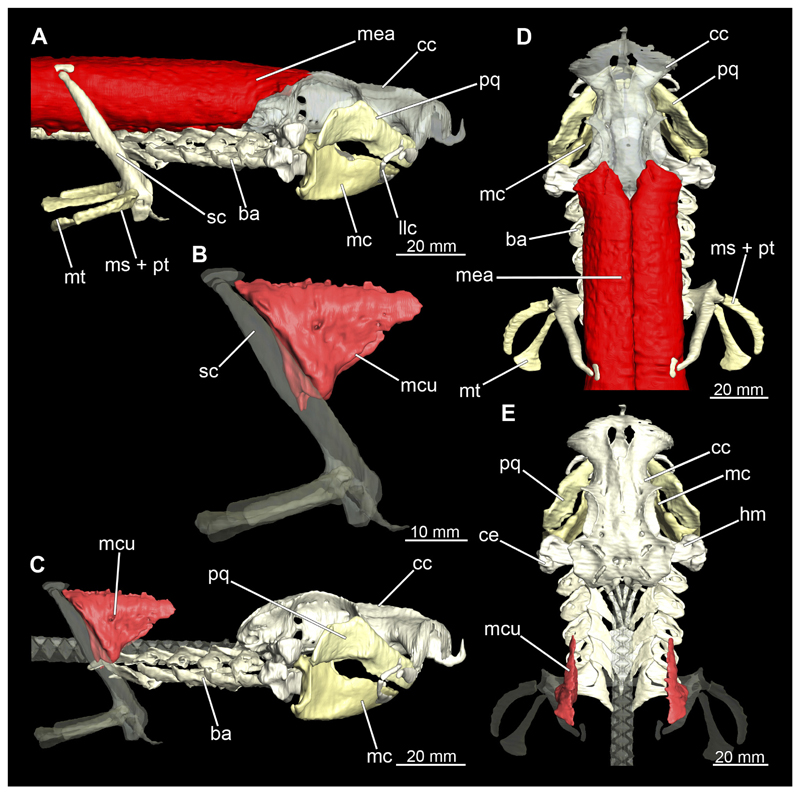
*Chiloscyllium punctatum*, female: Musculus epaxialis and Musculus cucullaris with pectoral girdle, visceral, hyoid and mandibular arches, and chondrocranium. **A** epaxialis lateral, **B** detail pectoral girdle with M. cucullaris lateral, **C** M. cucullaris lateral, **D** M. epaxialis dorsal and **E** M. cucullaris dorsal view as a rendered surface. Abbreviations: ba, branchial arches; cc, chondrocranium; ce, ceratohyal; hm, hyomandibula; llc, lower labial cartilage; mc, Meckel’s cartilage; mcu, musculus cucullaris; mea, musculus epaxialis; ms, mesopterygium; mt, metopterygium; pq, palatoquadratum; pt, pteropterygium; sc, scapula.

The pectoral fins, which articulate laterally with the shoulder girdle, consist of three cartilages at their base, with several radialia attached distally (Figs. 40B and 42B). The basal cartilages are from anterior to posterior the propterygium, mesopterygium and metapterygium (Figs. 41F, 42B, 43F, 44B). The propterygium is rather small and flat. In the micro-CT scan, the protopterygium is indistinguishable from the mesopterygium and both appear fused (Figs. 39F and 41F). In the dissected specimens, however, the protopterygium can be distinguished as a separate (very fine) cartilage (Figs. 40B and 42B). Only one radialia attaches to the protopterygium. The mesopterygium is positioned between the protopterygium and the metapterygium. It is sickle-shaped and curved caudally (Figs. 39F, 40B, 41F, 42B). The metapterygium is about the same length as the mesopterygium, but broadens distinctly distally, ending in a spatulate widening. The radialia are generally tripartite and comprise a longer proximal, a shorter median and a shorter distal component.

All components of the shoulder girdle are very similar in both sexes and hardly or not at all different. In the male specimen, only the foramina for the pectoral nerves and artery are not identifiable in the micro-CT models, but this is due to the resolution of the scans, as it is clearly visible in the dissected shoulder girdle.

#### 3.2.10 Muscles associated with the pectoral girdle

##### 3.2.10.1 Musculus cucullaris

The Musculus cucullaris is positioned between the M. epaxialis and the Musculus constrictor branchiales superficial (Figs. 28, 30, 43, 44). It is a relatively thin but planar muscle. The M. cucullaris originates from the fascia of the M. epaxialis and attaches to the dorsolateral surface of the posterior two epibranchialia and to the scapular process of the pectoral girdle.

##### 3.2.10.2 Pectoral fin musculature

**Figure 45:**
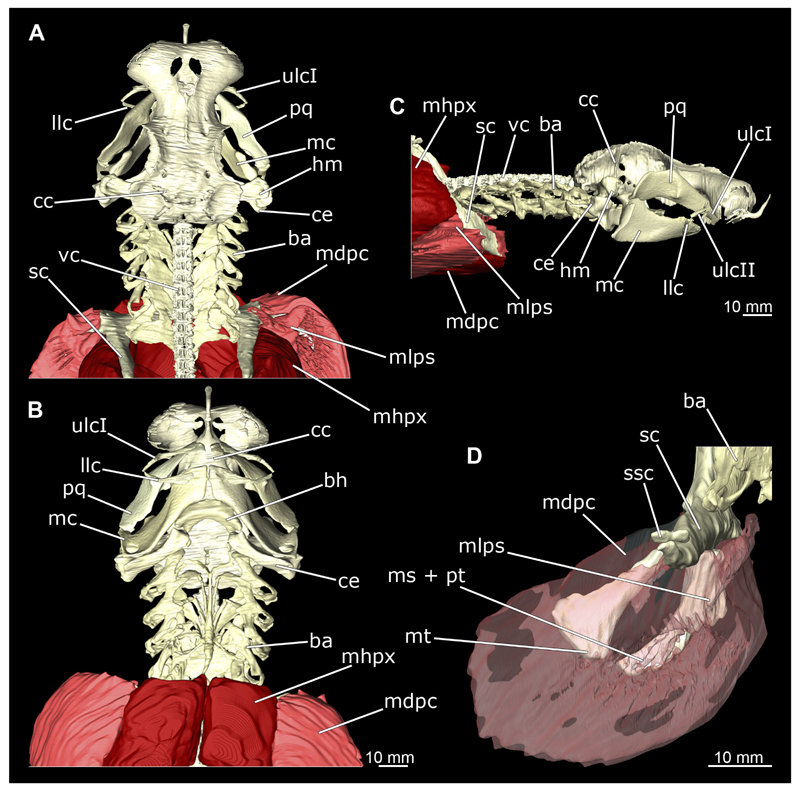
*Chiloscyllium punctatum*, male: Musculus hypaxialis and muscles of the pectoral fin, visceral, hyoid and mandibular arches, vertebral column and chondrocranium. **A** dorsal, **B** ventral, **C** lateral and **D** detail of the pectoral fin dorsal views as a rendered surface. Abbreviations: ba, branchial arches; bh, basihyale; cc, chondrocranium; ce, ceratohyal; hm, hyomandibula; llc, lower labial cartilage; mc, Meckel’s cartilage; mdpc, musculus depressor pectoralis; mhpx, musculus hypaxialis; mlps, musculus levator pectoralis superficialis; ms, mesopterygium; mt, metopterygium; pq, palatoquadratum; pt, pteropterygium; sc, scapula; ssc, suprascapula; ulcI, upper labial cartilage I; vc, vertebral column.

**Figure 46:**
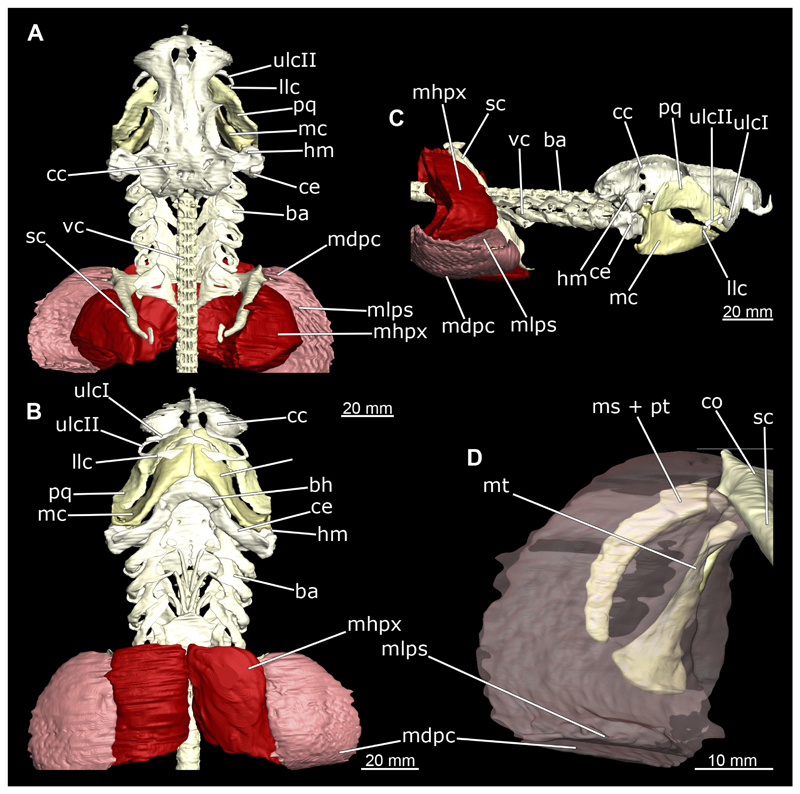
*Chiloscyllium punctatum*, female: Musculus hypaxialis and muscles of the pectoral fin, visceral, hyoid and mandibular arches, vertebral column and chondrocranium. **A** dorsal, **B** ventral, **C** lateral and **D** detail of the pectoral fin dorsal views as a rendered surface. Abbreviations: ba, branchial arches; bh, basihyale; cc, chondrocranium; ce, ceratohyal; co, coracoid; hm, hyomandibula; llc, lower labial cartilage; mc, Meckel’s cartilage; mdpc, musculus depressor pectoralis; mhpx, musculus hypaxialis; mlps, musculus levator pectoralis superficialis; ms, mesopterygium; mt, metopterygium; pq, palatoquadratum; pt, pteropterygium; sc, scapula; ulcI, upper labial cartilage I; ulcII, upper labial cartilage II; vc, vertebral column.

The dorsal musculature of the pectoral fin consists essentially of the Musculus levator pectoralis, which comprises one superficial and two inferior layers (Figs. 45A,C–D and 46A,C–D). The M. levator pectoralis superficialis is relatively thick and covers the proximal half of the dorsal side of the fin in a fan shape (Goto 2001). This muscle originates from the lateral side of the scapula. The Musculus levator pectoralis inferioris is short and lies below the M. levator pectoralis superficialis on the base of the dorsal side of the pectoral fin (Goto 2001). Its origin is at the Processus levator pectoralis and inserts at the basal cartilages of the pectoral fin. The Musculus levator pectoralis distalis is relatively narrow, also fan-shaped and starts distal to the M. levator pectoralis inferioris at the distal edges of the basal cartilages of the pectorals and ends at the base of the proximal radialia (Goto 2001). The ventral component of the pectoral musculature is the Musculus depressor pectoralis (Figs. 45 and 46). This is relatively massive and also fan-shaped. It originates from a fossa lateral to the coracoid and attaches to the ventral surface of the fin.

#### 3.2.11 Heart

**Figure 47:**
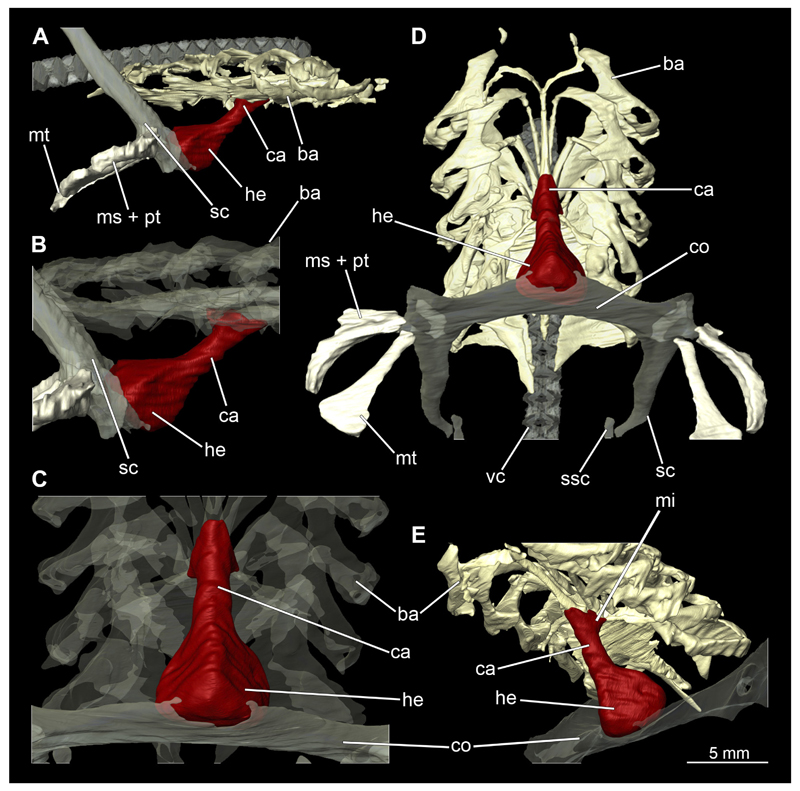
*Chiloscyllium punctatum*, male: Different views of the heart and neighbouring cartilaginous structures. **A** lateral, **B** detail lateral, **C** detail ventral, **D** ventral and **E** latero-ventral views as a rendered surface. Abbreviations: ba, branchial arches; ca, conzs arteriosus; co, coracoid; he, heart; ms, mesopterygium; mt, metopterygium; pt, pteropterygium; sc, scapula; ssc, suprascapula; vc, vertrebral column.

**Figure 48:**
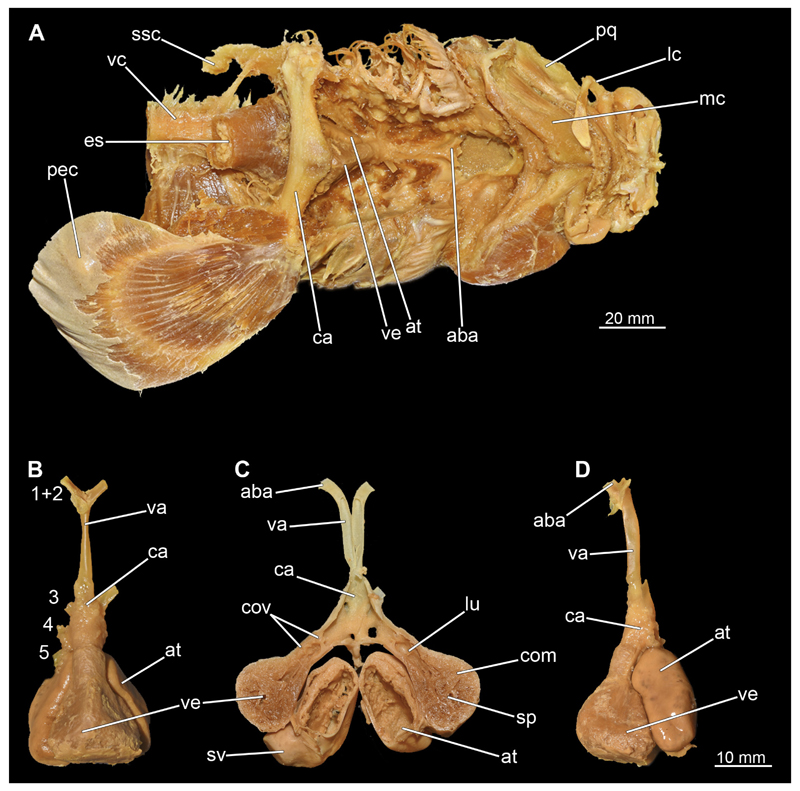
*Chiloscyllium punctatum*, male: Different views of the heart of the dissected specimen: **A** heart lying on the apron of the shoulder girdle ventral, **B** ventral, **C** sagittal cut and **D** lateral views of the dissected specimen. Abbreviations: 1–5, individual branchial arteries 1–5; aba afferent branchial arteries; at, atrium; ca, conus arteriosus; com, compacta; cov, conus valves; es, esophagus; lc, labial cartilage; lu, lumen; mc, Meckels ba, mc,Meckel’s cartilage; pec, pectoral fin; pq, palatoquadratum; sp, spongiosa; ssc, suprascapula; sv, sinus venosus; va, ventral aorta, vc, vetrebtal column, ve, ventricle.

**Figure 49:**
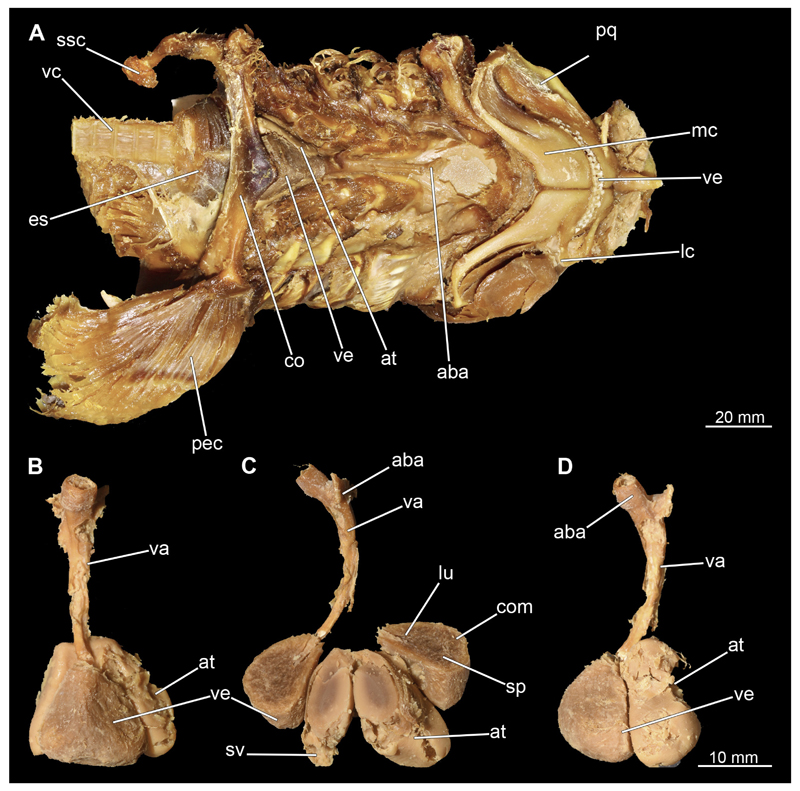
*Chiloscyllium punctatum*, female: Different views of the heart of the dissected specimen: **A** heart lying on the apron of the shoulder girdle ventral, **B** ventral, **C** sagittal cut and **D** lateral views of the dissected specimen. Abbreviations: 1–5, individual branchial arteries 1–5; aba afferent branchial arteries; at, atrium; ca, conus arteriosus; com, compacta; cov, conus valves; es, esophagus; lc, labial cartilage; lu, lumen; mc, Meckel’s cartilage; pec, pectoral fin; pq, palatoquadratum; sp, spongiosa; ssc, suprascapula; sv, sinus venosus; va, ventral aorta, vc, vetrebtal column, ve, ventricle.

The heart is located in the pericardium. This in turn is supported by the apron of the shoulder girdle (Figs. 47, 48A, 49A). Only in the male it was possible to reconstruct the heart from the micro-CT scan. As could be seen during the dissection, the iodine had attacked the heart tissue in the female quite severely, which is why it appeared very diffuse and unclear in the micro-CT data. In the micro-CT model of the male heart, however, relatively little detail can be seen. Atrium and ventricle appear as a fused unit (Fig. 47) and the boundaries cannot be determined. The only other element that stands out is the conus arteriosus. Much more detail is seen in the dissection of the heart (Figs. 48 and 49). Due to the lack of internal pressure, caused by the blood flow, the individual blood vessels are collapsed and are not easy to observe. In ventral view, the ventricle is the most prominent structure (Figs. 48 B and 49 B). It is a large, slightly pyramidal, muscular chamber. It rests on top of the atrium, which is only indistinctly visible in ventral view. Cranially, adjacent to the ventricle is the conus arteriosus, which is the fourth chamber of the shark’s heart and contains several rows of conus valves. Descending from the conus arteriosus are the individual branchial arteries (Fig. 48B). Gills III–V have their own artery, while gills I and II have a shared inflow through the anterior (afferent) branchial artery. The left lateral view nicely shows the position and size relationship of the atrium to the ventricle. The atrium is supplied by the two lateral common cardinal and the two caudal suprahepatic veins, which flow into the first ventricle that is the sinus venosus (Tota 1999). The sinus venosus lies mostly dorsal and its apex points towards the atrium, into which it flows via the sinoatrial junction. The sinus venosus is located on the right dorsal side and is clearly visible in Figure 48C and 49C in the longitudinal section of the heart. The atrium is connected to the ventricle via the atrioventricular valve. The compartmentation of the ventricle is clearly visible in the dissection (Figs. 48C and 49C). It has three basic areas. In the centre is the so-called spongiosa, which has a sponge-like appearance as it is built up of countless very thin muscle bundles (trabeculae). Surrounding the spongiosa is the compacta (especially ventrally). This is built up by dense, ordered muscle bundles, which give the tissue a much denser structure. According to Tota (1999) it is only found in a few teleosteans with high metabolic or locomotor activity, but in many chondrichthyans from various groups. Just anterior to the transition to the conus arteriosus is the so-called lumen. To get from the lumen to the conus arteriosus, the blood passes through several pairs of conal valves (Figs. 48C). From there, the bloodstream divides into the individual afferent branchial arteries, where it is oxygenated by flowing through the gills. The structure of the heart is equivalent in both sexes and differs only in the suboptimal condition of heart tissue preservation in the female.

#### 3.2.12 Vertebral column

**Figure 50:**
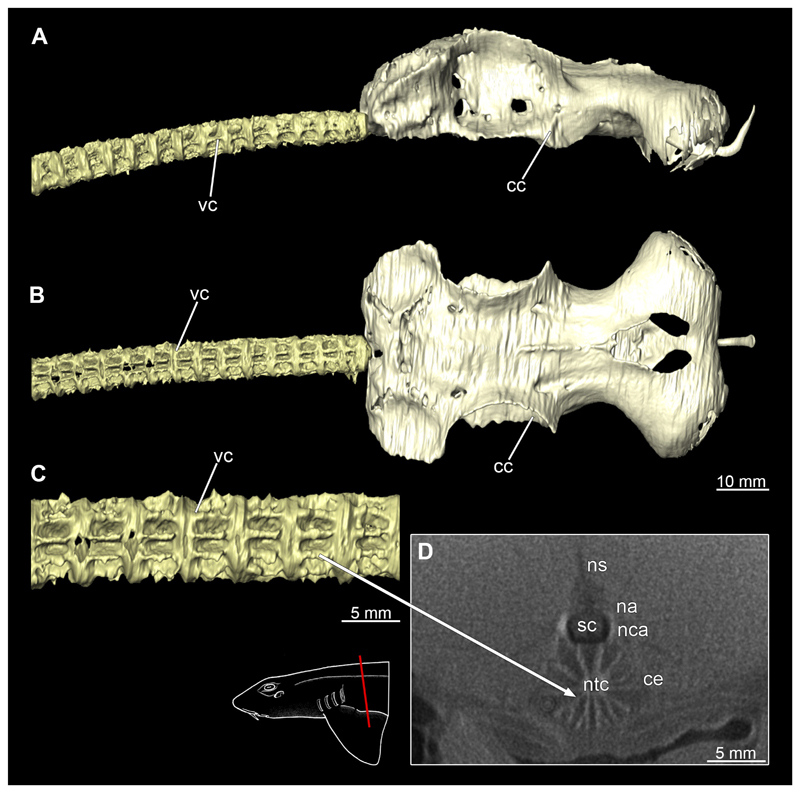
*Chiloscyllium punctatum*, male: Vertebral column with chondrocranium as rendered surface. Arrow pointing to the longitudinal ribs running around the centre in the CT-image. **A** lateral, **B** dorsal, **C** detail lateral and **D** transversal cross-section through dorsal region (CT-image). Abbreviations: cc, chondrocranium; ce, centrum; na, neural arch; nca, neural canal; ns, neural spine; ntc, notochord; sc, spinal cord; vc, vertebral column.

**Figure 51:**
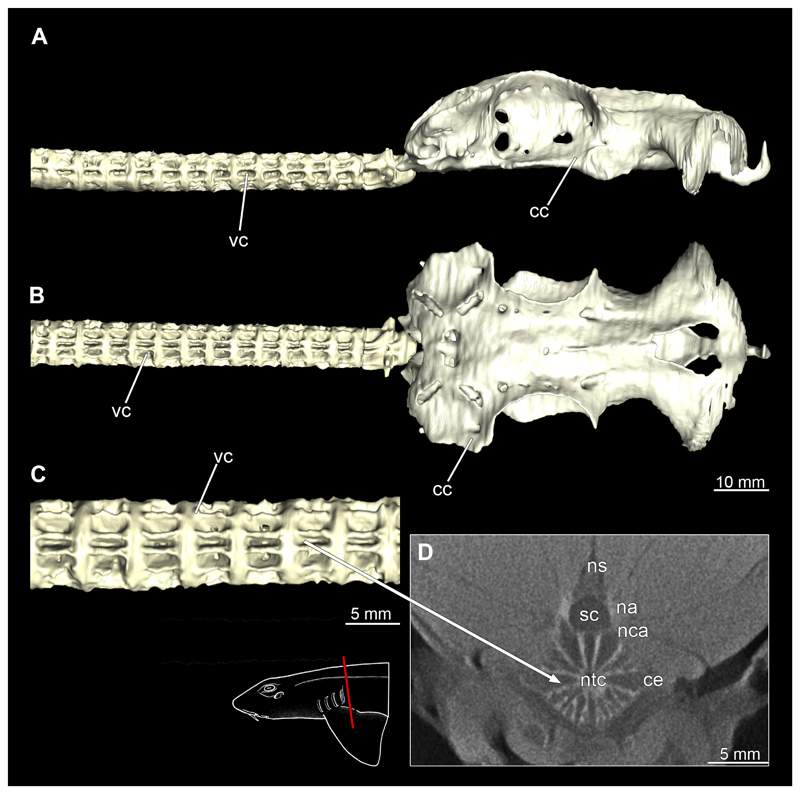
*Chiloscyllium punctatum*, female: Vertebral column with chondrocranium as rendered surface. Arrow pointing to the longitudinal ribs running around the centre in the CT-image. **A** lateral, **B** dorsal, **C** detail lateral and **D** transversal cross-section through dorsal region (CT-image). Abbreviations: cc, chondrocranium; ce, centrum; na, neural arch; nca, neural canal; ns, neural spine; ntc, notochord; sc, spinal cord; vc, vertebral column.

The vertebral column is the central supporting structure or cartilage staff in all vertebrates. In Chiloscyllium as in all chondrichthyans, the notochord is persistent and runs through a central canal of the vertebral centra (Figs. 50D and 51D). The vertebral column also provides a protective cover for the spinal cord, which runs through the spinal canal formed by the neural arches. The structure of the vertebrae can be seen very clearly in the micro-CT scans (Figs. 50 and 51). The neural arches continue dorsally into the neural spine, which serves as attachment points for the epaxial muscles. In the micro-CT scans, it was only possible to reconstruct the vertebral centra, as the neural spine or the neural arch hardly shows any calcification (Figs. 50A-C and 51A–C). Several longitudinal ribs run around the centre. These are terminated cranially and caudally of each vertebra by a flat plate. Both sexes have a similar vertebral morphology.

#### 3.2.13 Musculus epaxialis and Musculus hypaxialis

Lateral to the vertebral column are the two epaxial muscles, which are massive muscle strands that extend dorsally over a large part of the body length (Figs. 28A, 30A, 43A,D, 44A,D). They insert anteriorly on the chrondrocranium at the sphenopterotic ridge (Figs. 28A, 30A, 43A, 44A). Caudally, each lateral muscle cord splits into dorsal and lateral components (Goto 2001). Further anteriorly these components are indistinct from each other. The ventral separation from the hypaxial musculature is quite clear due to the horizontal myoseptum. The Musculus hypaxialis consists of only one muscle strand, which forms a very thin muscle layer, especially ventrally (Figs. 28A–C, 30A–C, 45A–C, 46A–C). This muscle envelops the abdominal cavity from the shoulder girdle to the pelvic girdle. Posteriorly, the muscle becomes again a longitudinal strand extending caudally (Goto 2001).

## 4 Discussion

### 4.1 Methodical discussion

The skeleton of chondrichthyans consists of cartilage, which is difficult to distinguish from the surrounding soft tissues using micro-CT scans. The different tissues therefore were enriched with iodine by submerging them in a staining solution. Iodine concentration occurred to different extent, depending on the density and composition of the corresponding tissue. Muscles, for example, absorb iodine easier than relatively dense cartilage. According to Jeffery et al. (2011), the differences in iodine accumulation in muscle compared to cartilage could be that the iodine is bound in the complex structure of glycogen within the muscle cells. This is because iodine and glycogen form a glycogen-iodine complex (Lecker et al. 1996). A persistent problem with staining objects preserved in ethanol is that the tissue dehydrates and thus shrinks relatively quickly when the object is stained in aqueous iodine solution. In addition, since the aqueous iodine solution penetrates the tissue more slowly than the alcoholic iodine solution, the sample remains in the solution much longer, which leads to even greater dehydration and thus quicker shrinkage. For this reason, the iodine was dissolved in ethanol to prevent this shrinkage as far as possible. If the sample would have remained in the solution longer, it might also have been possible to visualise other structures, such as tendons and ligaments. Extending the staining time would possibly also intensify the staining of individual nerves and thus improve their visibility in the CT scan. However, it is questionable whether intensified staining would have improved the visibility of fine structures, given the relatively low scanning resolution of 75 μm due to the size of the scanned sample. In the female, the double concentration of iodine was used for staining, but the staining time was 25 days less than for the male. The results show that the staining intensity of the two specimens is not significantly different. To determine up to which point further iodine enrichment is no longer possible, specimens would have to be scanned at regular intervals, which was impossible in this study. The severe tissue damage in the female specimen can clearly be attributed to the higher iodine concentration, as the other parameters were the same as during staining of the male specimen. The subcutaneous connective tissue and the tissue of the heart muscle seem to be particularly affected. No significant shrinkage was observed in both specimens, which is why a longer staining time should be preferred than using a higher iodine concentration.

### 4.2 Morphological discussion

*Chiloscyllium punctatum* (brownbanded bamboo shark) lives mainly in and around coral reefs, intertidal pools, flat water zones and reef walls (Ebert et al. 2016). As highly multi-layered habitats, coral reefs clearly represent the definition of a n-dimensional hypervolume. Bamboo sharks are highly adapted to these conditions. Their flat, elongated shape allows them to live in such intricate habitats, as they can fit through small openings in the reef and move with great manoeuvrability. With their wide, shovel-like rostrum, they are also able to move objects, such as stones or pieces of coral, around to reach food (MS, pers. obs.). The streamlined rostrum also makes it easier for them to search for prey deeper in the substrate. *Chiloscyllium punctatum* feeds mainly on bottom dwelling invertebrates and possibly small fishes (Ebert et. al 2021; Compagno 1984). According to Wilga et al. (2007), the closely related species *Chiloscyllium plagiosum* (Anonymous [Bennett], 1830) is an obligate suction feeder. Due to the very similar morphology, the close relationship of both species and observations of their feeding behaviours made in situ, the species studied here also can be considered an obligate suction feeder. The animals often dig for prey in the soft substrate, as described by Motta and Wilga (2001) for *C. plagiosum.* In doing so, the animals invade the sediment up to the level of the first gill slit and dig, e.g., for invertebrates with a combination of suction and head movements (MS, pers. obs.). The prey consequently is sucked into the oral cavity and the sand is expelled through the spiracles and especially the rearmost two gill slits. To make this type of feeding feasible, a water mass in front of the predator’s mouth has to be moved into the mouth. This has to happen fast enough, in order, together with the viscosity of the water, to prevent the prey from escaping (Wilga et al. 2007). If suction takes place close to any substrate (preferably solid), this can extend the distance of the suction effect, as a volume of water has to be moved, regardless of the spatial orientation of the same. Also helpful here is a dorsally located rostrum, which favours a precise direction of the suction jet (Nauwelaerts et al. 2008). These authors described that this type of feeding near a substrate, in combination with the rostrum, can increase the effective sucking distance of *C. plagiosum* by up to 2.5 times. The nurse shark *Ginglymostoma cirratum* (Bonnaterre, 1788) has an effective sucking distance of only about 3 cm in front of the mouth and due to the exponential decrease in effective sucking distance, these animals are probably not very effective active predators, but have to approach their prey, ambush them, or restrict themselves to sessile or slow prey (Motta et al. 2008). Again, striking close to the substrate can increase the range. According to Wilga et al. (2007), nurse and bamboo sharks are sometimes very similar in their feeding behaviours, which is why a similar hunting behaviour can also be assumed for *C. punctatum.* In orectolobiform sharks, the suction is mainly generated by the expansion of the pharyngeal cavity with the help of the very prominent and strongly developed hypobranchial musculature (Wilga and Sanford 2008). The main muscles involved are the M. coracoarcualis, M. coracohyoideus and M. coraco-branchialis (Motta et al. 2008). The existing hypertrophy of these muscle groups is even more indicative of their role as obligate suction feeders. According to common hypotheses, the food transport is produced by the coordinated movement of the hyoid and branchial arches as well as the shoulder girdle. In the study by Van Meer et al. (2019), it was shown that there are essentially several distinct individual phases of food movement within the pharynx. The main movement in the first stage is accomplished by the retraction of the hyoid arch and the closing of the mandibular arch. The effective food transport takes place through the generated water currents. In the stationary phase of the food that follows, there is probably no significant retrograde movement only because the food is retained by the branchial arches. A second water flow transports the food the remaining way to the oesophagus, but significantly slower than the first step. In the study, there was little correspondence between the movement of the shoulder girdle and the food.

However, the shoulder girdle undoubtedly plays an important role in feeding, locomotion, respiration and body circulation of animals (Gudo and Homberger 2002). It serves as an attachment point for the Muscoli epaxialis and hypaxialis, the M. cucullaris and the previously described hypobranchial muscles. In addition, the pericardium is supported and protected by the median apron of the shoulder girdle (Marinelli and Strenger 1959). The two pectoral fins attach to the shoulder girdle. These are special in *Chiloscyllium* insofar as, similar to *Hemiscyllium,* this shark uses them far more diversely than many other shark species. Typically, these two genera use their relatively flexible pectoral (and pelvic) fins to rest on them on the substrate and to move by performing a primitive crawl, rarely even above water. This mode of locomotion may be supported by a hypertrophied area of the Musculus hypaxialis, which attaches to the posterior part of the pectoral girdle at the scapular process. This point of attachment causes the scapula to be inclined or rotated caudally by the M. hypaxialis at this point. If the M. hypaxialis is only activated on one side and alternately on both sides of the body, it could have an even more beneficial effect on a “crawl”. In addition, the hypaxial muscle provides support for the shoulder girdle when the hypobranchial muscles located cranial to the pectoral girdle are contracted (Wilga and Lauder 2001). In addition, the hypaxial muscle provides support for the shoulder girdle when the hypobranchial muscles located cranial to the pectoral girdle are contracted (Wilga and Lauder 2001). These adaptations are also evident in the muscles of the fins themselves. These seem to be distinctly different from those of pelagic sharks, which use the fins mainly for manoeuvring. Wilga and Lauder (2001) state that bamboo sharks probably rely more on manoeuvrability than on a stable water position. In doing so, they use the principle of lifting wings to create vortices, which locally generate a partial negative pressure in the medium and give the animal lift or downforce. This also is the case when the animals rest on a substrate.

Usually, the fins are placed concave upward and are thus additionally pressed against the substrate by eddies. Especially in currents due to swell, tides, etc., this is to their advantage in their habitat. This also can be seen in animals kept in the aquaria (MS, pers. obs.). Depending on the intensity of the current, the animals stand more or less erected. The stronger the current, the more acute the angle to the bottom seems to be. Thus, the behavioural observations and the morphology studied match the studies of Wilga and Lauder (2001).

The sexes differ morphologically in only a few aspects according to the results of this study. Most visible are differences in the shape of the head, the chondrocranium and the mandibular arch. In dorsal view, the male specimen is much broader, especially in the middle to posterior region of the head. This is due to the enlarged Musculus adductor mandibulae and Musculus constrictor superficialis dorsalis/ventralis. Mating is probably induced after initial courtship behaviour by the male by biting into the female’s pectoral fin (Cornish 2005; Masuda 1998). In other species, after briefly continuing to swim parallel, both sexes take up a vertical position facing downwards. This was described for several shark species, some of them very closely related to the bamboo shark *(Triaenodon obesus* (Tricas and Le Feuvre 1985), *Ginglymostoma cirratum* (Pratt and Carrier 2001), *Hemiscyllium freycineti* (Cornish 2005)). *Chiloscyllium griseum* (Dral 1980) and *Hemiscyllium ocellatum* (West and Carter 1990) mate in a horizontal position. The mating behaviour of *C. punctatum* cannot be inferred with certainty from the behaviour of related species, as there seems to be a large variation within the group. However, the consensus is that the male has to fixate the female in all positions, which takes place, as mentioned before, by biting into the pectoral fins. The different morphologies of the male chondrocranium and the male jaw probably present adaptations to this behaviour. Especially the upper jaw in the male specimen shows clear morphological differences to that of the female one. The upper jaw of the male has a relatively long and straight labial edge. The chondrocranium also is elongated in this area (between the orbit and the nasal capsules) compared to the female, which also facilitates a longer extension of the jaw elements. In the symphyseal, the jaw describes a concave arc forming a hook-like protrusion, which is not developed in the female. If the male now takes the pectoral fin of the female into its mouth, it could use this extension to hold the anterior edge of the fin in place. The posterior part of the fin is relatively flexible and could easily bend and run anteriorly in the ellipsoidal recess between the two jaws. Additional support would be provided by the teeth of the upper and lower jaws holding on to the fin and the very rough skin of the pectoral fins themselves. As the available data on how the female is fixed by biting the pectoral fin seems to be rare, this process remains merely a hypothesis based on morphological features described herein. Whether seasonally different morphologies of the jaw and teeth occur, as is known from other cartilaginous fish species (e.g., Rangel et al. 2016), is questionable and cannot be clarified in this study. For this, several specimens have to be examined at different times of the year in order to be able to determine a change during the mating season with certainty. As the animals studied are clearly in a size range where they can be considered sexually mature, the theory that sexual dimorphic dentitions only occur at sexual maturity is not applicable (Chen and Liu 2006; Fahmi et al. 2021).

*Chiloscyllium punctatum* belongs to the orectolobiform sharks, which in turn are member of the galeomorph sharks. In addition to the Orectolobiformes, the group Galeomorphii includes three additional orders, i.e., Heterodontiformes, Lamniformes, and Carcharhiniformes (Compagno 1973), which are considered monophyletic. This monophyly is based, among other things, on the fact that in Galeomorphi the M. levator labii is directly connected to the neurocranium and not through a tendon as in basal representatives of the Squalomorphi. According to Carvalho (1996), the origin of the M. levator labii at the upper preorbital wall in Orectolobidae, *Heterodontus zebra* and Parascylliidae can be considered a synapomorphy of the galeomorphs (considered secondarily lost in the other galeomorph groups). Specifically, for *C. punctatum,* Luther (1909a) also reported this feature in his work. Also, in the two specimens examined here, the position of the muscle on the orbitonasal lamina and the dorsal side of the chondrocranium can be confirmed. Additionally, there were no signs of an attachment point via a tendon, but rather a firm, quite planar connection of the muscle, directly with the cartilage surface. As described by Soares and Carvalho (2013) for Heterodontiformes and Orectolobiformes, the M. levator labii attaches anteriorly to Meckel’s cartilage also in *Chiloscyllium* and thus only allows a small opening of the jaws. This was also observed in the sections of the specimens examined, but this muscle strand is only relatively thin, which is why it could not be detected in either of the two sexes in the micro-CT data. In the male specimen, however, the muscle protrudes slightly ventrally beyond the palatoquadratum and could thus indicate this muscle strand. In contrast, in Lamniformes and Carcharhiniformes, this muscle is located more posteriorly and is at least partially fused with the adductor mandibulae muscle, so that a larger mouth opening can be realised (Soares and Carvalho 2013).

Another feature of *C. punctatum* that has phylogenetic significance is the morphology of the oculomotor muscles. In all groups of the Orectolobiformes, there is an additional bundle of the M. rectus inferioris. However, with the exception of *Parascyllium* and *Cirrhoscyllium,* an extra bundle of M. rectus internus is present that is symmetrical to the extra bundle of M. rectus inferioris (Goto 2001). As this feature was clearly visible in both sexes in the manual dissection, it confirms Goto’s (2001) description and differentiates *C. punctatum* easily from *Parascyllium* and *Cirrhoscyllium.*

The sense of sight is generally highly developed in elasmobranchs and is an important element of the hunting strategies in many species. In general, elasmobranchs have a firm cartilaginous sclera around the eye and a robust cornea anteriorly. The cornea has the same refractive index as salt water, so it does not affect the quality of vision. The lens is generally large and free of chromatic aberrations. On the posterior side, in addition to the retina, there are the choroidea and the suprachoroidea. In the examined *C. punctatum* specimen examined here, little detail of the visual sense is detectable in the micro-CT data, except for the relatively large, round lens. In general, due to the natural habitat of *C. punctatum,* one could conclude that the sense of sight is well developed in shallow waters, which is exposed to a high light flux, and plays an important role in hunting. However, since the animals prefer to be active at dusk or even night, this hypothesis can be rejected. Behavioural observations showed that the animals hardly hunt visually, but rely primarily on their other senses, especially the sense of smell (Hodgson and Mathewson 1971; Mathewson and Hodgson 1972).

The sense of smell seems to play a particularly important role in the life of this species, especially in obtaining food. Chemical stimuli are the dominant stimuli towards food for many sharks (Hodgson and Mathewson 1971). When observing the animals, it is immediately noticeable that they react mainly to scents. For example, food in the water is not visually targeted by these sharks, but by moving the head back and forth to identify the direction of the prey item by its smell (MS, pers. obs.). The animals seem to practice klinotaxis like the nurse sharks in the study by Mathewson and Hodgson (1972). It can also be ruled out that electroreception by Ampullae of Lorenzini is used, which would locate moving prey as shown by feeding experiments in aquaria using dead fishes as food items. Compared to *Squalus acanthias,* the nasal capsules are distinctively larger and so is the space for the olphactoric sacs, in which the olfactory receptors are located (see also Shirai, 1992, fig. 5.1). This suggests that the sense of smell in *C. punctatum* either plays a more important role than in *S. acanthias,* or *S. acanthias* has more efficiently folded epithelium within the nasal capsules to obtain the same, or possibly a larger, receptive surface area. The nostrils are specifically shaped to form a lateral inflow and a proximal outflow opening. This ensures optimal circulation through the olphactoric sacs. As mentioned above, the inside of the olphactoric sacs is folded in a very complex way, which leads to a considerable increase in their surface area. The sense of smell seems to be even more important when considering the habitat in which the animals live. Smells can travel very far in water. The reef as a complex structure with crevices, channels and open passages has the effect that such olphactoric stimuli are channelled and can be targeted via the animals’ stereo perception. The shark can even easily detect prey buried in the sand through its sense of smell, as experiments conducted by the first author have shown.

What has also been repeatedly observed is the response of sharks to auditive stimuli (Davies et al. 1963). Inner ears of sharks that rely on a distinctly raptorial life-style show a much higher development of sensory epithelia and structures associated with a dorsal sound channel than sharks that tend to rely on more immobile prey. An ear that is strongly adapted for hearing prey appears to provide a selective advantage, e.g., for carcharhinids (Corwin 1989). Sharks do not have a pressure/displacement transducer in the inner ear but are presumably only capable of perceiving the displacement components of acoustic waves as stimuli (Maisey 2001). Nevertheless, various studies have shown that sharks are in principle capable of semi-directional hearing (Tester et al. 1972; Corwin 1977). Due to the properties of water, hearing is only possible for fishes in the range up to ca. 1000 Hz, as the frequencies above this are transmitted poorly or not at all (Maisey 2001; Parmetier et al. 2019). The thousands of sensory hair cells located in the lower part of the inner ear form the sensory macula (Lowenstein and Roberts 1951; Maisey 2001). Within this macula there are granules known as otoliths. (Corwin 1989). They act as an inertial mass to stimulate the hair cells (Parmetier et al. 2019). No otoliths were found in either the micro-CT scans or the section. As they represent at least partially crystalline structures, they would be particularly noticeable in the micro-CT images as distinct elements within the inner ear (Schnetz et al. 2019). Since the specimens studied here were alcohol-fixed, the otoliths probably dissolved. Few studies on otoliths from cartilaginous fishes are known so far, as they are rarely found. This could be related to the fact that these loose granulates mostly consist of phosphate containing crystals (with components of collagen), which are only weakly bound and thus do not represent a solid, mineralised structure. In an alcoholic medium, the otoliths apparently decompose easily, which led to the fact that sharks and rays were long thought not to have them (Schnetz et al. 2019). The dorsal endolymphatic duct seems to be also evident for the mechanoreception, i.e., the sense of balance of the animals (Maisey 2001). For example, an occlusion of the duct leads to the fact that they (can) lose orientation and balance (Portmann 1921). The vestibular organ is capable of perceiving acceleration, which gives the sharks particular agility and precision during hunting (Lowenstein and Roberts 1951). Young (1981) described in his work that fishes exposed to slower turning speeds have larger semicircular canals than fast swimming fishes. The slower the rotational movement, the more sensitive the sensory equipment must be to detect the resulting forces. Fast-swimming species tend to have narrow semicircular canals with a relatively small inner radius, as the forces and thus sensory stimuli that occur can be many times higher and the sensory cells must therefore be less sensitive to perceive the necessary stimuli (Young 1981). Bamboo sharks can also swim relatively fast, but generally have a rather sedate lifestyle and are not so much designed to chase prey as to stalk and track it. Their semicircular canals are also relatively extended, which would be in accordance with Young’s (1981) theory of the morphological adaptation of the vestibular organ to the lifestyle.

## 5 Conclusions

This study is the first to describe the morphology of an orectolobiform shark species in detail using both manual dissection and micro-CT data. This has been done with particular attention to possible morphological differences between male and female specimens to establish possible sexual dimorphic patterns for the first time in the cranium of orectolobiform sharks. Dimorphic differences between the sexes can be observed in the specimens investigated. In order to substantiate these observations with further data, additional specimens of both sexes and other species should be included in future studies.

The now digitally available content of the dissections and the micro-CT reconstructions will serve as a basis for further studies and should be a first step for a series of comparative studies on the cranial morphology of orectolobiform sharks including establishing cranial sexually dimorphic features.

## Figures and Tables

**Figure 12:**
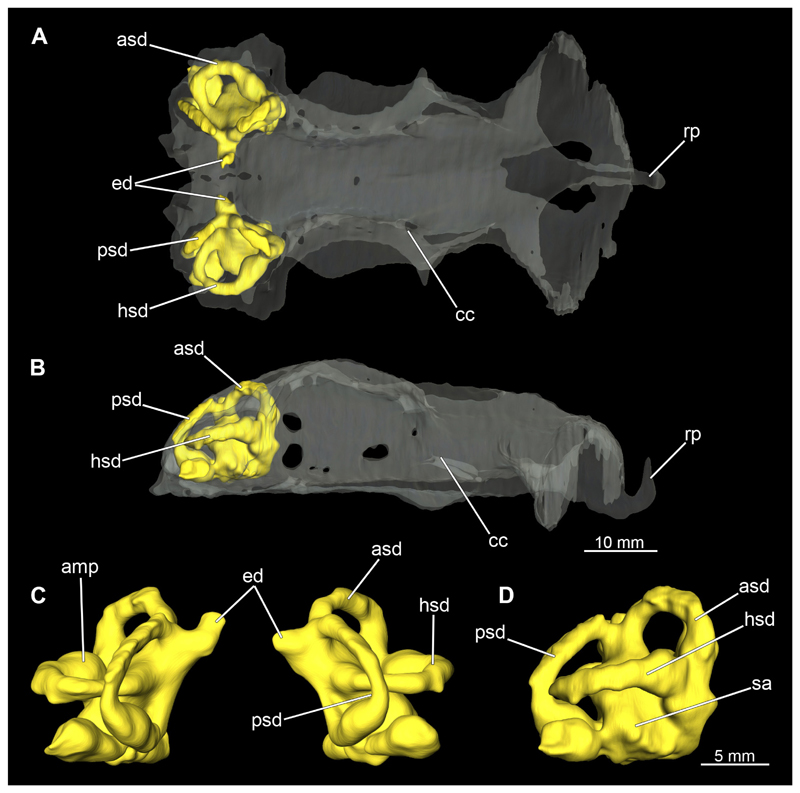
*Chiloscyllium punctatum*, female: Different views of the inner ear of the specimen. **A** dorsal view within the chondro-cranium, **B** lateral view within the chondrocranium, **C** posterior view and **D** lateral view as rendered surface. Abbreviations: amp, Ampulla; asd, anterior semicircular canal, cc, chondrocranium; ed, endolymphatic duct; hsd, horizontal semicircular canal, psd, posterior semicircular canal, rp, rostral process; sa, sacculus.

**Figure 32:**
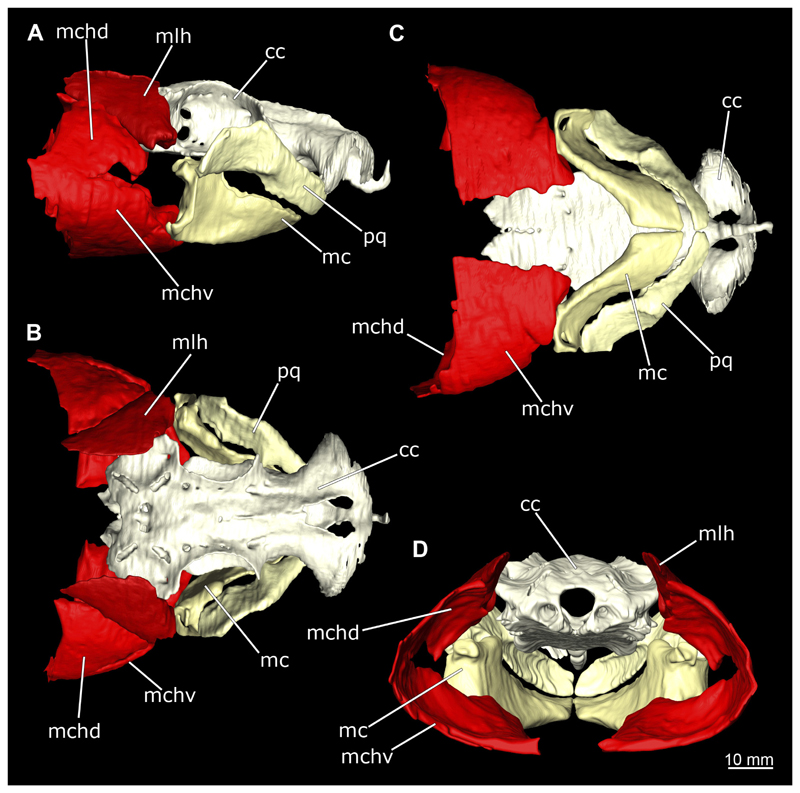
*Chiloscyllium punctatum*, female: Different views of the postcranial muscles, together with the Chondrocranium and the mandibular and hyoid arch of the specimen. **A** lateral, **B** dorsal, **C** ventral and **D** posterior views as rendered surface. Abbreviations: bh, basihyale; cc, chondrocranium; ce, ceratohyal; hm, hyomandibula; mam, musculus adductor mandibulae; mc, Meckel’s cartilage; mchd, musculus constrictor hyoideus dorsalis; mchv, musculus constrictor hyoideus ventralis; mim, mlh, musculus levator hyomandibulae; pq, palatoquadratum.

**Table 1:** Protocol for preparation for staining

Storing solution	80% EtOH (96%)+ 20% Deionat
0 h	66% EtOH (96%) + 33% Deionat
24 h	33% EtOH (96%) + 66% Deionat
96 h	100% Deionat
168 h	100% EtOH (96%) + 50 g / 100 g I_2_ (s)

**Table 2:** Overview over the used scanning parameters for the CT scans of the male and female of *Chiloscyllium punctatum*

Description	Days after immersion in the staining solution	kV	mA	Exposition time [ms]	Voxelsize [mm]	Filter	Number of images
Male	172	130	320	1400	0.075	0,75mm copper filter	2204
Female	147	150	230	1400	0.075	0,5mm copper filter	1923
